# Lack of expression of miR-29a/b1 impairs bladder function in male mice

**DOI:** 10.1242/dmm.050054

**Published:** 2023-06-07

**Authors:** Zunyi Wang, Robert Spitz, Chad Vezina, Jianghui Hou, Dale E. Bjorling

**Affiliations:** ^1^ Department of Surgical Sciences, University of Wisconsin-Madison, Madison, WI 53706, USA; ^2^ The O'Brien Center for Urologic Research, University of Wisconsin-Madison, Madison, WI 53706, USA; ^3^ Comparative Biosciences, University of Wisconsin-Madison, Madison, WI 53706, USA; ^4^ Division of Nephrology, Washington University School of Medicine, St Louis, MO 63110, USA; ^5^ Urology, University of Wisconsin-Madison, Madison, WI 53706, USA

**Keywords:** miR-29a, Bladder fibrosis, Uroflow, Cystometry, Mice

## Abstract

Lower urinary tract symptoms (LUTS) refer to various urological diseases, and incomplete bladder emptying is common among affected patients. The etiology of LUTS is largely unknown, and investigations of LUTS suggest that bladder fibrosis contributes to pathogenesis of LUTS. MicroRNAs (miRNAs) are short (∼22 nucleotides), non-coding RNAs that repress target gene expression by a combination of mRNA degradation and translation inhibition. The miR-29 family is best known for its anti-fibrotic role in various organs. miR-29 was decreased in bladders of patients with outlet obstruction and a rat model of bladder outlet obstruction, suggesting that miR-29 may contribute to impaired bladder function subsequent to tissue fibrosis. We characterized bladder function in male mice lacking expression of *Mir29a* and *Mir29b-1* (miR-29a/b1). Lack of miR-29a/b1 resulted in severe urinary retention, increased voiding duration and reduced flow rate, and these mice failed to void or voided irregularly during anesthetized cytometry. Collagens and elastin were increased in bladders of mice lacking miR-29a/b1. These findings reveal an important role for miR-29 in bladder homeostasis and suggest the therapeutic potential of miR-29 to improve symptoms in patients with LUTS.

## Teaser

**Summary:** Male mice lacking microRNA-29a/b1 develop bladder enlargement and diminished bladder contractility, associated with upregulation of genes related to extracellular matrix formation and turnover.

## INTRODUCTION

Lower urinary tract symptoms (LUTS) refer to a spectrum of urological disorders. These are generally divided into storage (urgency, frequency and nocturia), voiding (slow stream, hesitancy and dribbling) or post-micturition symptoms (a feeling of incomplete emptying), based on their temporal relationship to the micturition cycle (Andersson, 2003; Chapple et al., 2008; Thomas and Abrams, 2000). LUTS significantly impair quality of life, and incomplete bladder emptying is common among these patients. Incomplete bladder emptying results from inability of the detrusor muscle to adequately contract to completely empty the bladder, with or without increased bladder outlet resistance, and is often defined urodynamically as detrusor underactivity. The etiology of LUTS is largely unknown, and the pathophysiological processes underlying LUTS are likely multi-factorial. Although LUTS affect all ages, the prevalence of LUTS increases with age (Al-Zoubi et al., 2022; Andersson, 2003; Chapple et al., 2008). Evidence from epidemiologic, clinical and basic research suggests that bladder fibrosis may be an important contributing factor to pathogenesis of LUTS (Deveaud et al., 1998; Lepor et al., 1992; Mirone et al., 2004). Fibrosis alters bladder architecture and increases stiffness of the bladder wall, changes that inhibit expansion and contraction of the bladder as it fills to store urine or contracts to expel urine. It was the consensus of a recently convened panel of clinicians and scientists that bladder fibrosis is present in most benign bladder disorders and that there is an acute need for more effective strategies to prevent and treat bladder fibrosis (Fry et al., 2018).

MicroRNAs (miRNAs) are a group of ∼22 nucleotide-long non-coding RNAs that repress target gene expression by a combination of mRNA degradation and inhibition of translation (Ekman et al., 2016; He et al., 2013). It is estimated that miRNAs regulate most human genes, and a significant number of disorders may arise because of malfunction of miRNA signaling (Friedman et al., 2009; Paul et al., 2018). Binding of miRNA to mRNA is directed by the seed region of the miRNA. The miRNA seed region is composed of the six to seven contiguous nucleotides starting at the second nucleotide (g2) from the 5′ end. Extensive investigations support the concept that the miRNA seed sequence determines target recognition and modulates miRNA targeting efficacy and specificity (Salomon et al., 2015; Schirle et al., 2014). Disrupting seed complementarity diminishes repression of target RNA (Lai, 2002; Lewis et al., 2005; Lim et al., 2005). However, the short length of the seed region allows relatively promiscuous binding of a single miRNA to multiple transcripts.

Although the functions of many miRNAs remain largely unidentified, the miR-29 family is best known for its anti-fibrotic role in various organs (Cushing et al., 2011; Noetel et al., 2012; van Rooij et al., 2008), and miR-29 has been referred to as a ‘master regulator’ of fibrosis (O'Reilly, 2016). The miR-29 family consists of *MIR29A* (miR-29a), *MIR29B1* (miR-29b1), *MIR29B2* (miR-29b2) and *MIR29C* (miR-29c), encoded by two distinct genomic clusters, miR-29a/b1 and miR-29b2/c. Mature miR-29s are highly conserved in human, mouse and rat (Ekman et al., 2016; He et al., 2013). The genes encoding miR-29a and miR-29b1 precursors are found on chromosome 6 in mice, while the genes encoding miR-29b2 and miR-29c in mice are on chromosome 1 (Ge et al., 2019; Ma et al., 2022). It has been demonstrated that miR-29b1 and miR-29a, as well as miR-29b2 and miR-29c, are transcribed together as polycistronic primary transcripts (Chang et al., 2008; Mott et al., 2010). The three miR-29 isoforms share identical sequences at nucleotide positions 2-7, the seed region that binds to the 3′ untranslated region of target mRNAs (Ekman et al., 2016; Harmanci et al., 2017; He et al., 2013; Kriegel et al., 2012). Recently, miRNA-sequencing analysis revealed that, although all three miR-29 isoforms are expressed across many mouse tissues, miR-29a is the dominant isoform, accounting for more than 50% of total miR-29 levels in most tissues analyzed (Caravia et al., 2018).

Interestingly, miR-29 is downregulated in the bladders of aging mice (Kim et al., 2017), patients with outlet obstruction (Gheinani et al., 2017) and a rat model of bladder outlet obstruction (Ekman et al., 2013), findings indicating that decreased miR-29 accompanies bladder fibrosis and impaired bladder function**.** It has also been reported that mice lacking expression of miR-29a/b1 develop bladder enlargement (Caravia et al., 2018). However, there is a lack of information regarding the effects of deletion of miR-29a/b1 on bladder structure and function. The present study was performed to test the hypothesis that systemic lack of miR-29a/b1 causes increased bladder fibrosis and impaired bladder function in male mice.

## RESULTS

### Bladder remodeling as a result of lack of miR-29a/b1

**Fig. 1.**
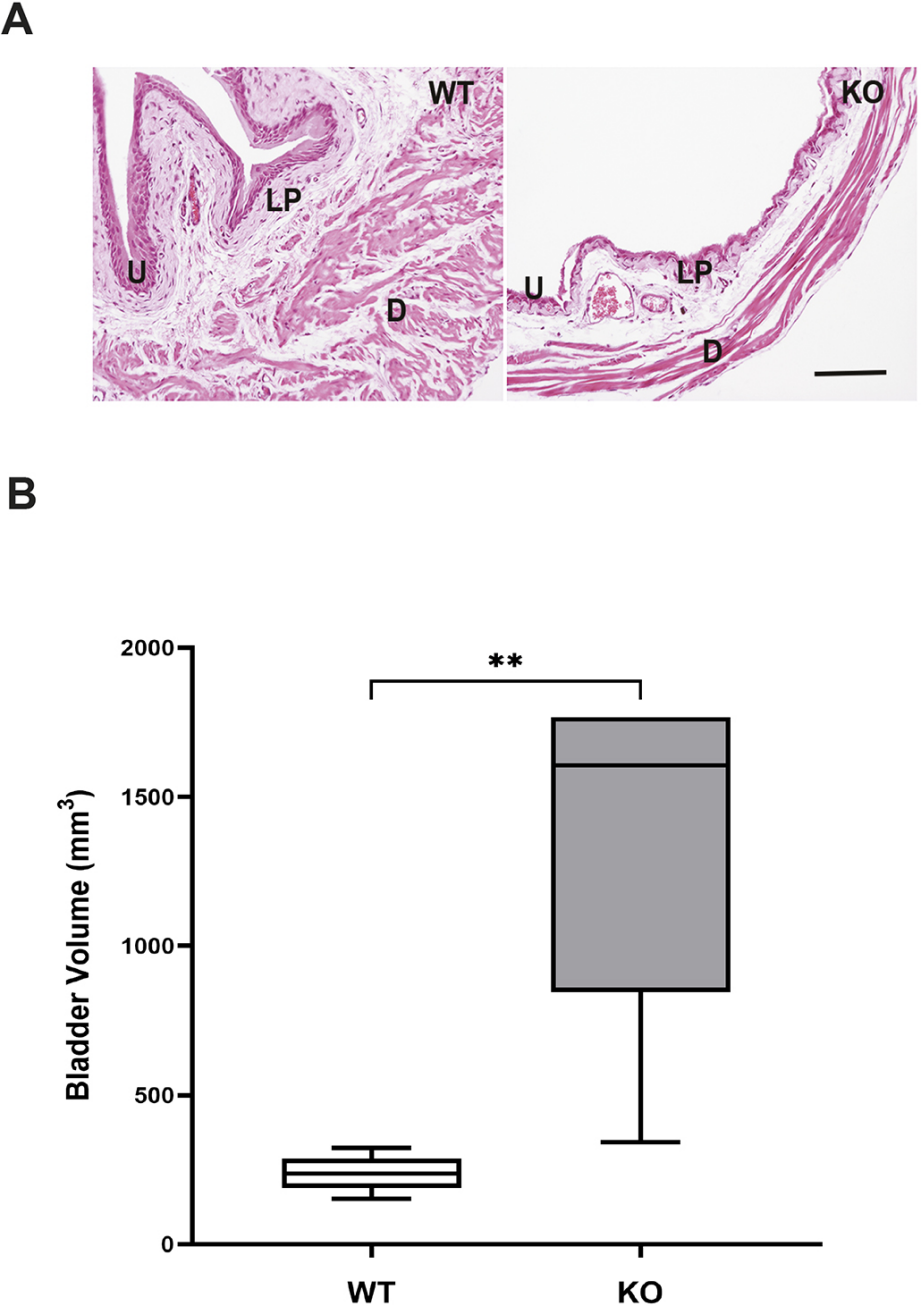
**Histological appearance of bladders and bladder volume.** (A) Representative Hematoxylin and Eosin (H&E)-stained images of wild-type (WT) and knockout (KO) bladders. D, detrusor; LP, lamina propria; U, urothelium. (B) Summarized data indicate significant enlargement of bladder volume in KO mice compared to that in WT mice. Data are shown as box and whiskers plot; center line is the median of the dataset, box limits indicate upper and lower quartiles, and whiskers show data minimum and maximum. *n*=6 WT and *n*=8 KO. **P<0.001 KO versus WT (unpaired two-tailed Student's *t*-test).

Reverse transcription PCR (RT-PCR) analysis of total bladder RNA confirmed that the miR-29a precursor was present in all wild-type (WT) bladders examined (*n*=8), but miR-29a precursor was below the detection limit in all knockout (KO) bladders (*n*=8), confirming lack of expression of miR-29a in KO mice. Consistent with a previous report (Caravia et al., 2018), age-matched KO mice weighed significantly less than WT mice (24±0.6 g, *n*=12 versus 31±1.3 g, *n*=11, respectively; *P*<0.01). Gross anatomical observation indicated that bladders of KO mice were significantly distended, with remarkable urinary retention, and had a much thinner wall than those of WT mice (Fig. 1A). The bladder volume of KO mice was nearly sixfold greater than that of WT mice (Fig. 1B), indicative of significant bladder distention because of lack of miR-29a/b1 expression.

### Altered urination pattern in conscious KO mice

**Fig. 2.**
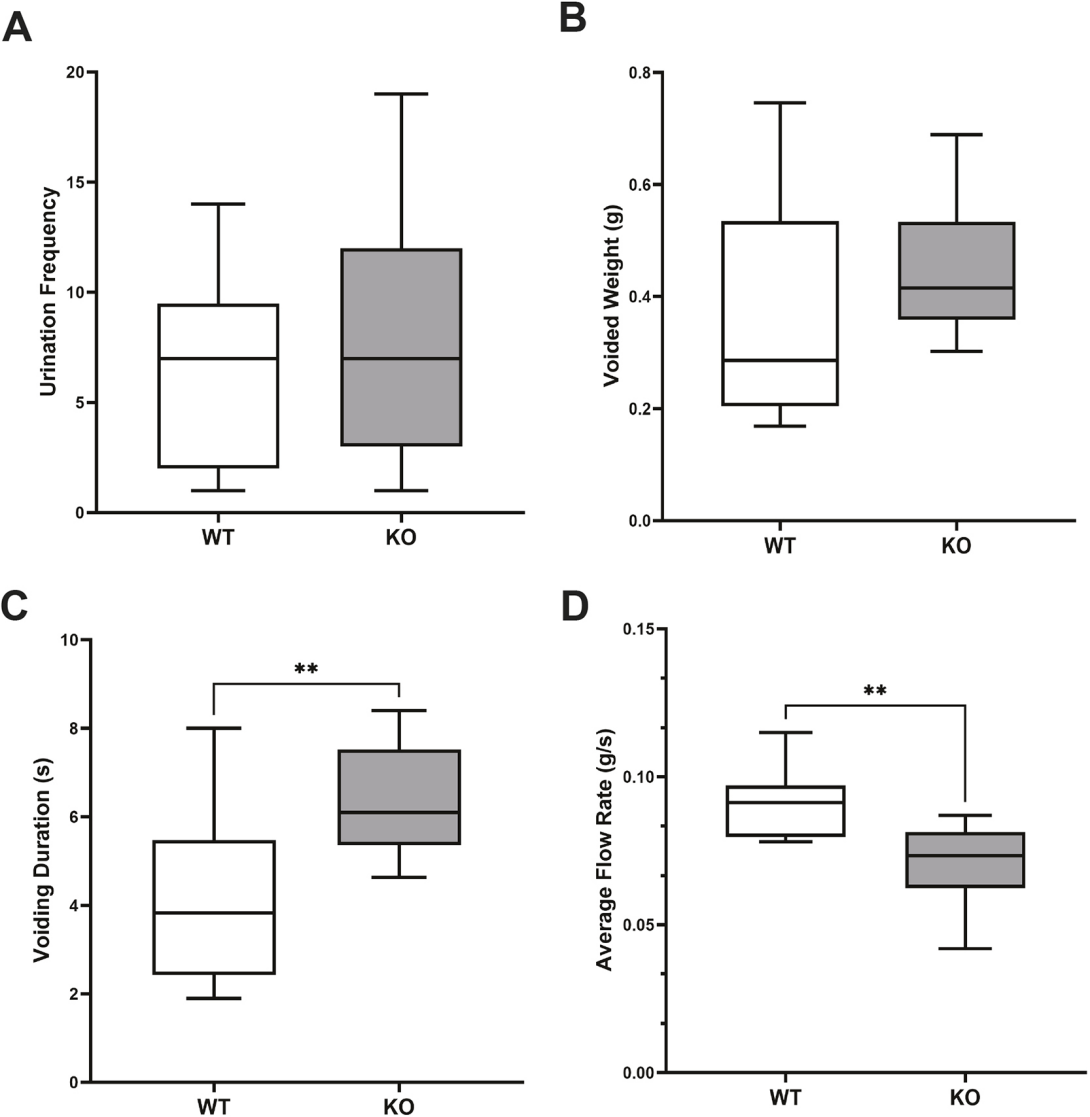
**Results of uroflowmetry.** (A-D) Summarized data of uroflowmetry indicate altered voiding pattern in KO mice. Urination frequency (A) and voided weight (B) were similar in WT and KO mice. The voiding duration (C) was increased, and flow rate (D) was reduced, in KO mice compared to WT mice. Data are shown as box and whiskers plots; center line is the median of the dataset, box limits indicate upper and lower quartiles, and whiskers show data minimum and maximum. *n*=9 WT and *n*=12 KO. **P<0.01 KO versus WT (unpaired two-tailed Student's *t*-test).

Bladder function was assessed in awake KO and WT mice by first evaluating voluntary voiding behavior in conscious WT and KO mice using uroflowmetry. WT mice typically exhibited several sustained voids per test session (Movie 1). In contrast, all KO mice exhibited voids that consisted of droplets of urine (Movie 2). Interestingly, urinary frequency was similar in WT and KO mice, and the weight of voided urine (indicative of voiding volume) and voiding frequency were not different between groups (Fig. 2A,B); however, voiding duration was significantly longer in knockout mice, indicative of reduced flow rate (Fig. 2C,D).

### Failure of regular voiding in KO mice during anesthetized cystometry

**Fig. 3.**
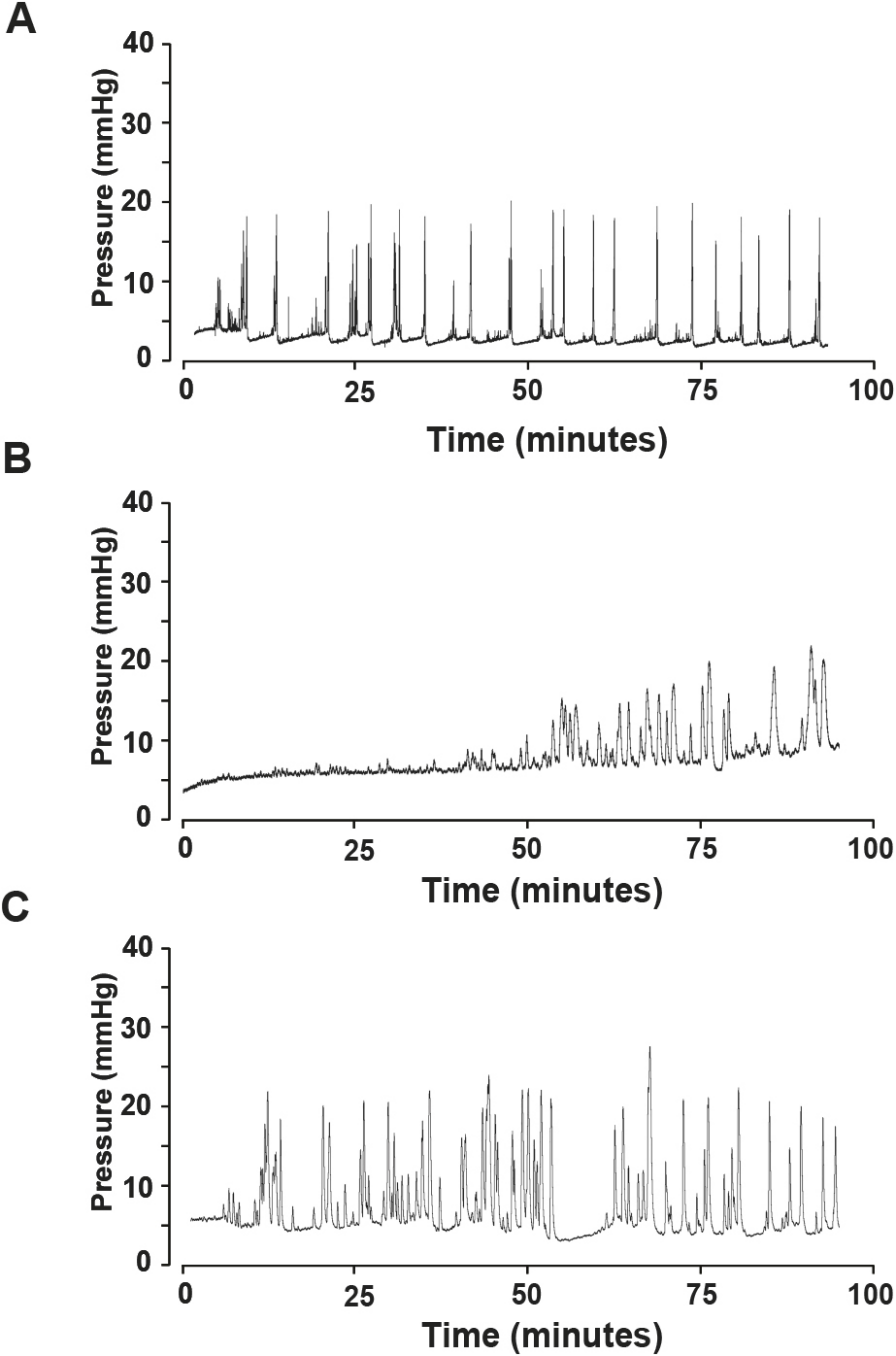
**Representative cystometrogram tracings of WT and KO mice.** (A) All WT mice had regular voids. (B,C) KO mice either had difficulty expelling urine through the urethra until a dribbling pattern characteristic of overflow was observed (B), or produced voids characterized by a series of contractions with release of droplets of urine and incomplete bladder emptying (C). *n*=5 WT and *n*=7 KO.

We next examined bladder function in anesthetized mice by performing continuous flow cystometry. Cystometry measures intravesical pressure during infusion of normal saline and determines several parameters associated with all phases of the micturition process. In response to saline infusion, all WT mice voided at regular intervals (Fig. 3A), similar to previously reported observations (Bjorling et al., 2015; Kim and Hill, 2017). In contrast, varying phenotypes were observed in KO mice, consistent with abnormal voiding physiology. In fact, four of seven KO mice had difficulty expelling urine during 1 h of saline infusion, and their bladders became overdistended (Fig. 3B). The other three KO mice that underwent anesthetized cystometry produced voids characterized by a series of contractions with release of droplets of urine, which were not considered to be complete voids, because bladder pressures did not return to baseline after contractions and release of urine (Fig. 3C).

### Decrease in electrical field stimulation (EFS)-induced responses in KO bladders

**Fig. 4.**
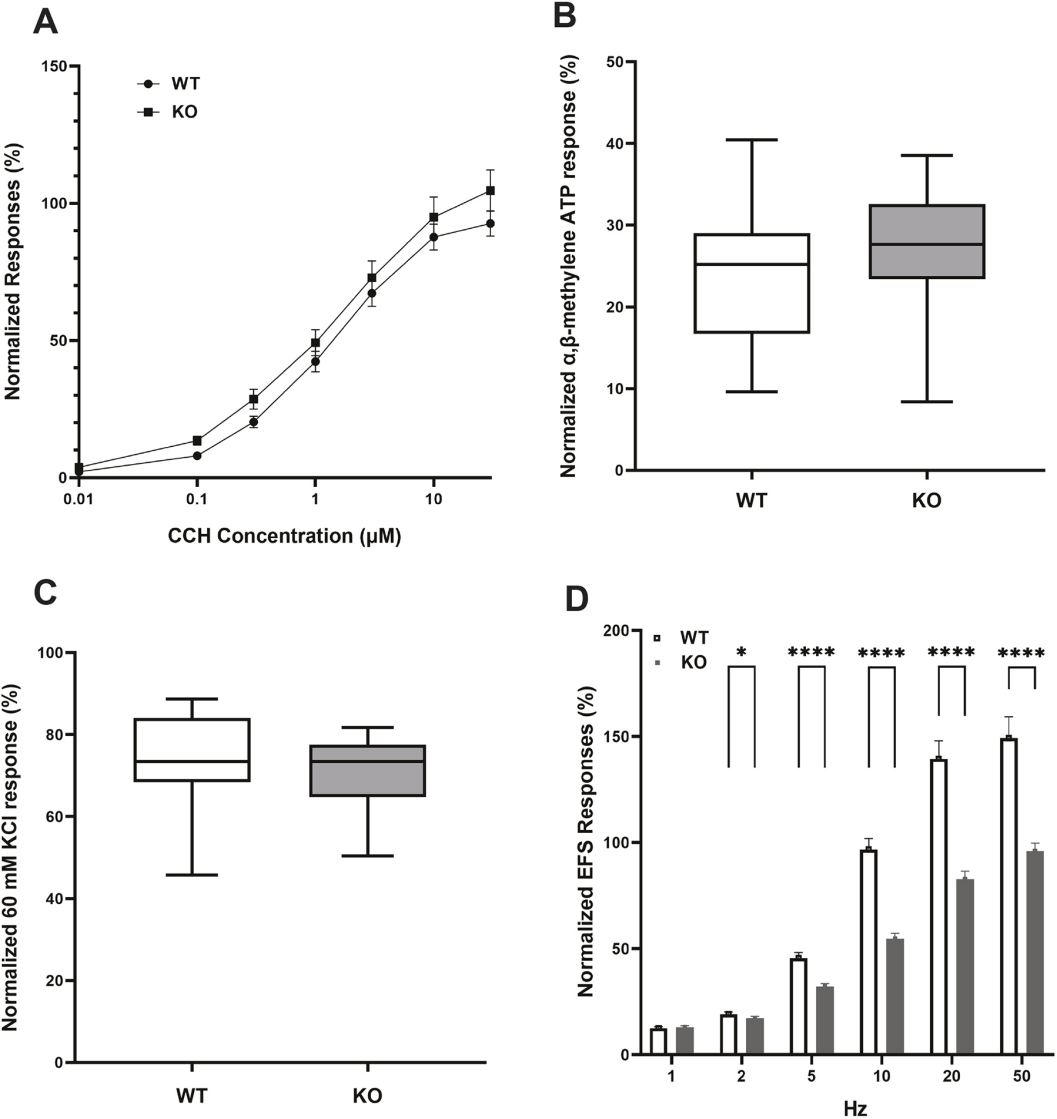
***In vitro* contractility of bladder tissue.** (A-D) KO bladders maintained contractile responses to carbachol (CCH; A), α,β-methylene ATP (B) and 60 mM KCl (C), but had impaired responses to electrical field stimulation (EFS; D), compared to WT bladders. Bars in A and D indicate s.e.m. Data in B and C are shown as box and whiskers plots; center line is the median of the dataset, box limits indicate upper and lower quartiles, and whiskers show data minimum and maximum. *n*=12 preparations from six mice of each genotype. *P<0.05, ****P<0.001, KO versus WT (unpaired two-tailed Student's *t*-test).

During micturition, bladder contractions are primarily induced by the neurotransmitters acetylcholine and ATP released from parasympathetic nerves (Andersson and Arner, 2004). We sought to determine whether impaired micturition in KO mice was related to decreased detrusor smooth muscle contractility. We first evaluated responses of isolated bladder strips to increasing concentrations of the cholinergic agonist carbachol (CCH). No significant differences in contraction in response to CCH were observed between WT and KO mice (Fig. 4A). In addition, responses to the purinergic receptor P2X agonist α,β-methylene ATP (10 µM; Fig. 4B) and to KCl (60 mM; Fig. 4C), which induces bladder smooth muscle contractions by direct depolarization of cell membranes (Vasquez et al., 2017), were comparable between WT and KO mice. These findings indicate that, even though bladders of Mir29 KO mice were distended compared to those of WT mice, the intrinsic contractile properties of the detrusor muscles of bladders were the same between genotypic groups.

EFS allows evaluation of neurally mediated contractile responses of isolated tissue strips, and EFS typically induces contractions of bladder strips in a frequency-dependent manner (Yu et al., 2018). EFS was applied at frequencies ranging from 1 to 50 Hz. The magnitude of response to EFS was lower in KO bladders than in WT bladders at 2, 5, 10, 20 and 50 Hz (Fig. 4D), indicating impaired neurotransmission in KO bladders.

### Differential gene expression analyzed with RNA sequencing (RNAseq)

**Fig. 5.**
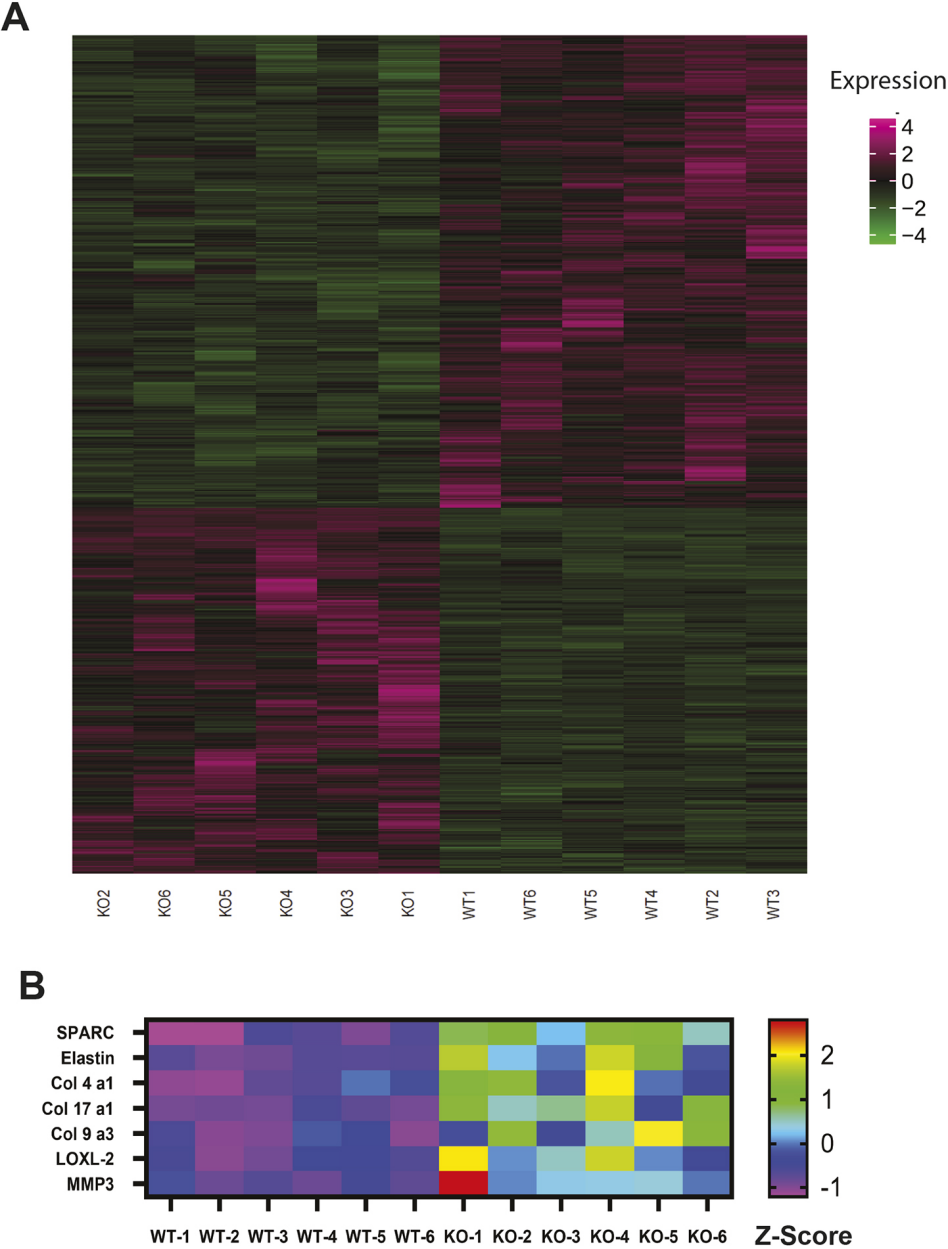
**Heatmaps showing all genes differentially expressed in bladders of KO mice compared to those of WT mice, and expression of genes associated with extracellular matrix upregulated in KO mice compared to WT mice.** (A) Heatmap illustrating all genes that were overexpressed (magenta) or underexpressed (green) in bladders of KO mice compared to those of WT mice. (B) Heatmap presenting upregulated genes related to extracellular matrix in bladders of KO mice compared to those of WT mice.

**Fig. 6.**
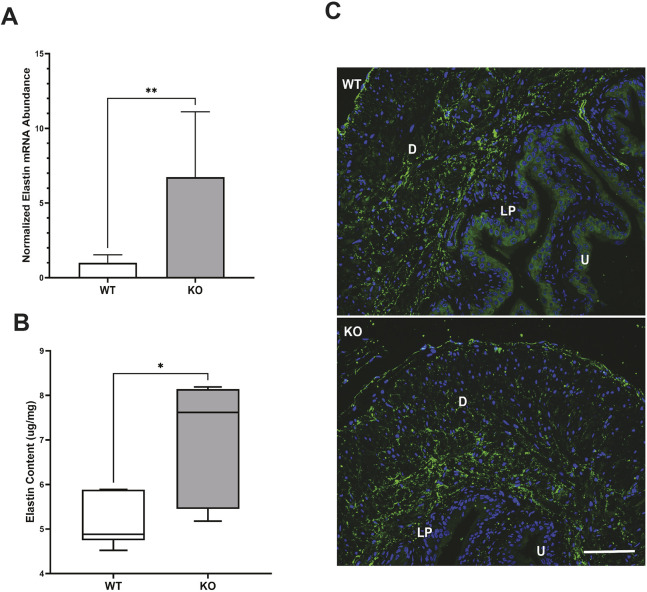
**Abundance of elastin mRNA and protein in bladders of KO and WT mice.** (A) RT-PCR revealed a significant increase in elastin mRNA abundance in bladders of KO mice compared to those of WT mice. Bars indicate s.e.m. *n*=8 KO versus *n*=8 WT. (B) Increased elastin protein content in the detrusor layer of bladders of KO mice compared to those of WT mice. Data are shown as box and whiskers plot; center line is the median of the dataset, box limits indicate upper and lower quartiles, and whiskers show data minimum and maximum. *n*=6 KO versus WT. *P<0.05, **P<0.01, KO versus WT (unpaired two-tailed Student's *t*-test). (C) Representative immunostaining image of elastin in the bladder wall. Green staining indicates elastin, and blue (DAPI) staining identifies nuclei. D, detrusor; LP, lamina propria; U, urothelium. Scale bar: 100 µm.

**Fig. 7.**
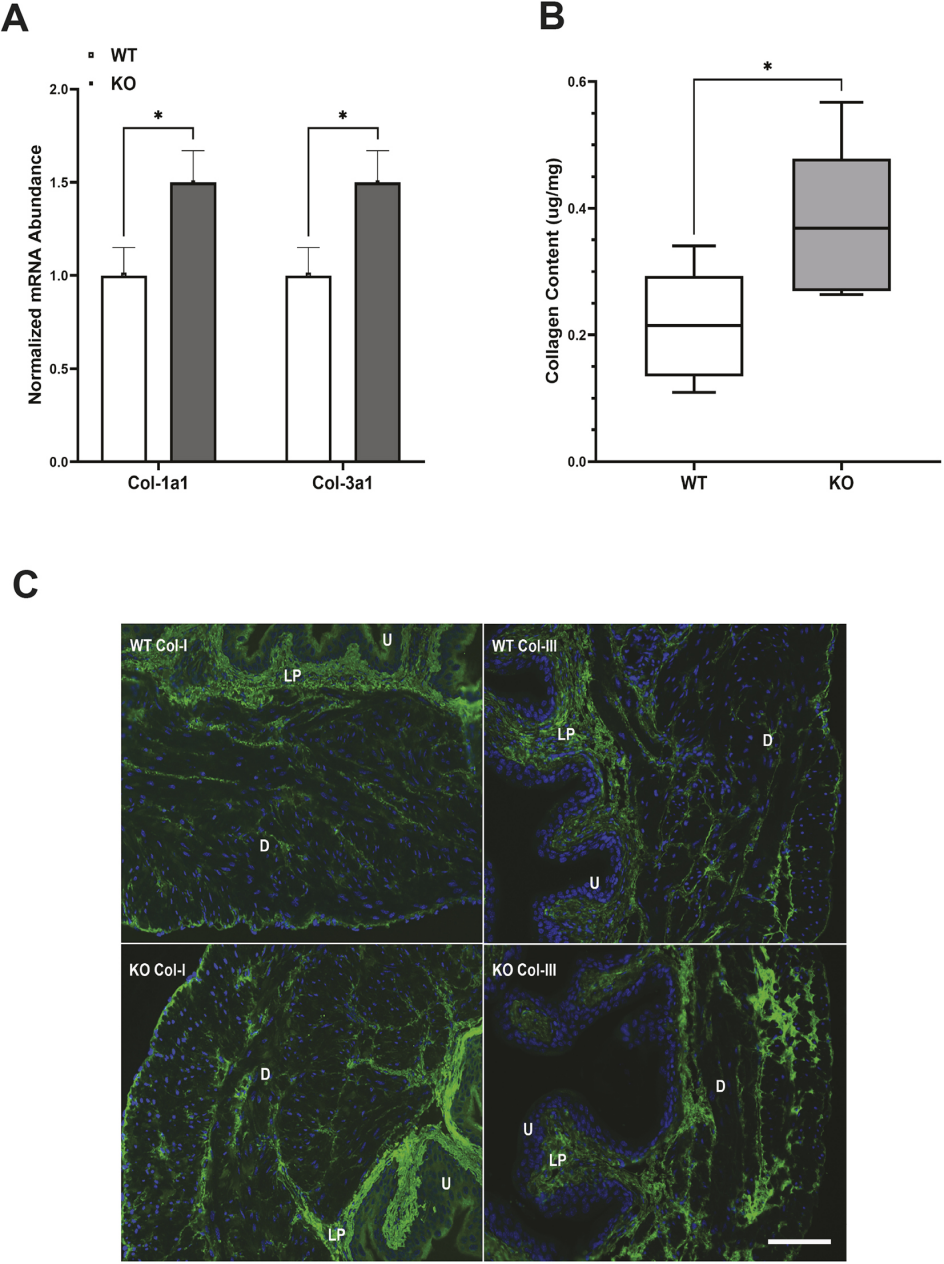
**Abundance of collagen 1a1 and 3a1 mRNA and collagen I and III protein in bladders of KO and WT mice.** (A) Real-time RT-PCR revealed a significant increase in collagen 1a1 and 3a1 mRNA abundance in bladders of KO mice compared to those of WT mice. Bars indicate s.e.m. *n*=8 KO versus WT. (B) Increased total collagen protein content in the detrusor layer of bladders of KO mice compared to those of WT mice. Data are shown as box and whiskers plot; center line is the median of the dataset, box limits indicate upper and lower quartiles, and whiskers show data minimum and maximum. *n*=6 KO versus WT. *P<0.05, KO versus WT (unpaired two-tailed Student's *t*-test). (C) Representative immunostaining of collagen I and III in the bladder wall. Green staining indicates collagen I or III, and blue (DAPI) staining identifies nuclei. D, detrusor; LP, lamina propria; U, urothelium. Scale bar: 100 µm.

We used RNAseq to identify genes that were differentially expressed between WT and KO bladders. We were interested in genes with the potential to affect bladder remodeling and cause physiological dysfunction in KO mouse bladders, particularly potential targets of miR-29 as predicted by TargetScan. RNAseq analysis demonstrated a remarkable consistency of gene expression in both WT and KO groups (Fig. S1, Table S1). To focus on the most biologically relevant changes while reducing the number of false positives, differential expression was determined using a threshold log_2_(1.5) fold-change cutoff. This resulted in 329 significantly upregulated and 425 downregulated genes when results from bladders of KO mice were compared with those obtained from bladders of WT mice (Fig. 5A). Elastin, a direct target of miR-29 and a key element of bladder extracellular matrix (ECM) (Ekman et al., 2013), was among the top 50 most upregulated genes in KO bladders (Fig. 5B). Additional predicted miR-29 targets that were upregulated included collagen IV and secreted protein acidic and rich in cysteine (SPARC). Expression of several other ECM molecules was also increased (Fig. 5B). Quantitative RT-PCR confirmed a nearly sixfold increase in elastin message in the KO bladders relative to WT bladders (Fig. 6A). In addition, RT-PCR also revealed a moderate, but significant, increase in expression of collagen 1a1 and 3a1 in the KO bladders (Fig. 7A). Data generated by RNAseq were analyzed using the Kyoto Encyclopedia of Genes and Genomes (KEGG) database to identify differentially regulated pathways in bladders from KO mice compared to those from WT mice (Kanehisa et al., 2023), and data were further interrogated using Database for Annotation, Visualization and Integrated Discovery (DAVID) to assess gene ontology (Huang et al., 2007). These analyses revealed multiple potential pathways that were differentially regulated in bladders from KO mice relative to bladders from WT mice. (Pathways identified by analysis of gene ontology that were upregulated in bladders from KO mice are shown in Fig. S1, and those that were downregulated are presented in Fig. S2.) It is interesting to note that 17 genes associated with the ECM within the cellular component were upregulated in bladders of KO mice. This is consistent with the previously identified role for miR-29 to suppress expression of components of the ECM. In contrast, multiple genes regulating ion transporters and channels, particularly those associated with potassium movement and signaling, were downregulated in RNA extracted from KO bladders.

### Collagen and elastin contents are increased in the detrusor of KO mice

Measurement of elastin (Fig. 6B) and total collagen (Fig. 7B) protein content confirmed increased presence of these ECM molecules in the bladders of KO mice (relative to bladders of WT mice), indicative of fibrosis in the KO bladders. Immunofluorescence demonstrated that elastin was mostly present in the detrusor and surrounded smooth muscle cells and muscle bundles (Fig. 6C). Collagen I and III (Fig. 7C) were present in the lamina propria and surrounded smooth muscle cells and muscle bundles. Immunofluorescence supported the observation of increased abundance of elastin, collagen I and collagen III in the bladders of KO mice compared to those of WT mice.

### Increased collagen abundance in prostatic urethra of KO mice

**Fig. 8.**
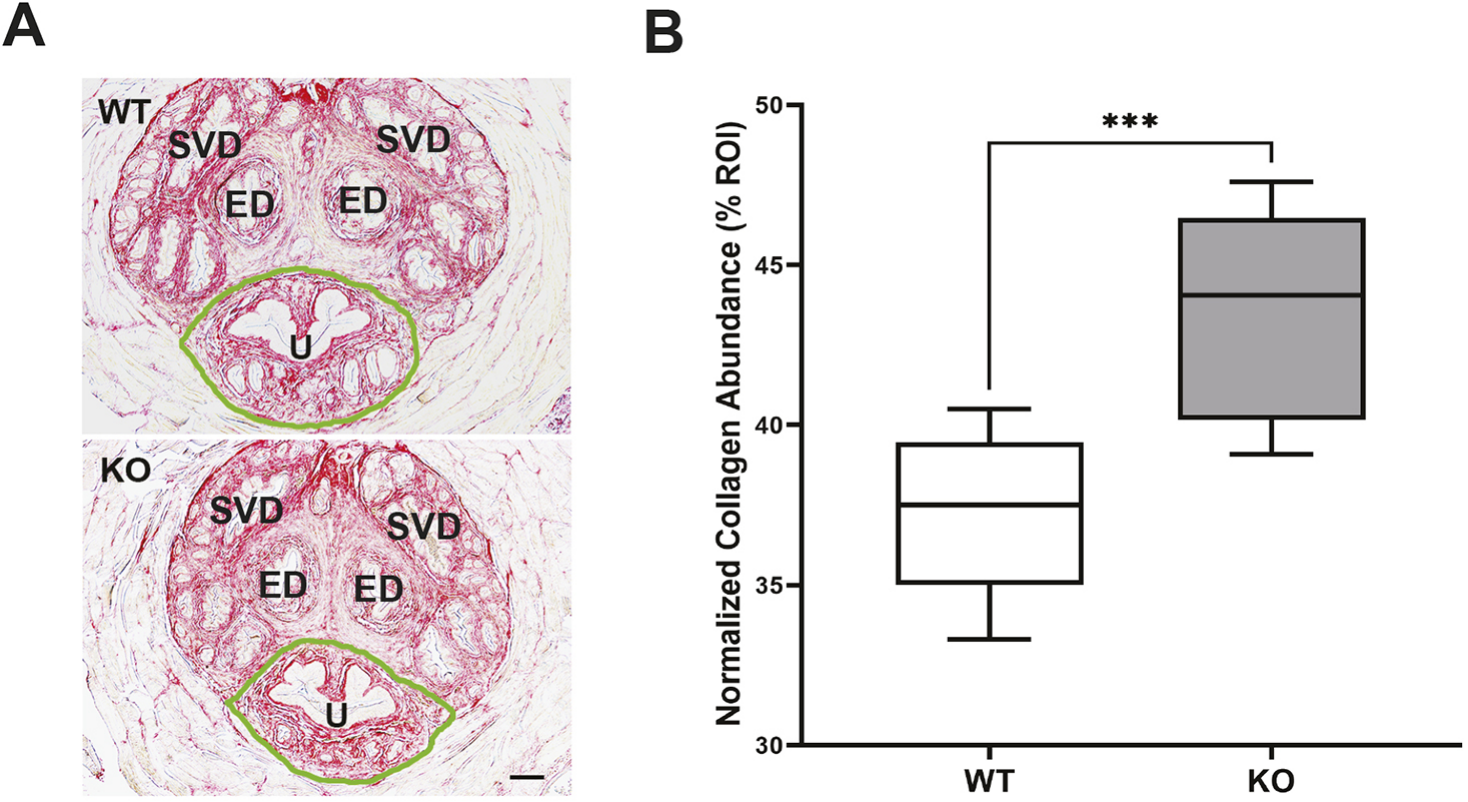
**Picrosirius Red staining of prostate tissues indicated increased collagen in the peri-urethral area.** (A) Representative Picrosiriuis Red (PSR) staining of the prostate. Collagen is detected by red staining, and the region of interest (ROI), including urethra (U), is outlined by the green line. ED, ejaculatory ducts; SVD, Seminal vesicle ducts. The light pink tissue surrounding the ducts and prostatic urethra is the rhabdosphincter. (B) Increased collagen protein content in the ROI of KO mice, compared to that of WT mice. Data are shown as box and whiskers plot; center line is the median of the dataset, box limits indicate upper and lower quartiles, and whiskers show data minimum and maximum. *n*=9 WT and *n*=10 KO. ***P<0.001, KO versus WT (unpaired two-tailed Student's *t*-test). Scale bar: 100 µm.

In patients with benign prostate hyperalgesia as well as in hormone-induced obstruction and accelerated aging animal models, prostatic fibrosis has been identified as a contributing factor to urinary dysfunction (Macoska et al., 2019; Nicholson et al., 2012). Evaluation of Picrosirius Red (PSR)-stained prostatic periurethral tissues (Fig. 8A; region of interest outlined in green) showed that the average collagen abundance was 17% greater in the prostatic urethra of KO mice, which was significantly different from that observed in WT mice (Fig. 8B). This observation, in conjunction with the observation of increased bladder size, fibrosis of the bladder wall and diminished *in vitro* response to EFS, supports the hypothesis that increased periurethral collagen within the prostate limits the capacity of urethra to relax during micturition process (Nicholson et al., 2012).

## DISCUSSION

Constitutive absence of miR-29a/b1 was accompanied by increased fibrosis of the lower urinary tract. KO mice also exhibited changes in voiding function and *in vitro* bladder contractility commonly associated with bladder fibrosis (Gheinani et al., 2017; Grover et al., 2011; Howard et al., 2004; Kamei et al., 2018). The *in vitro* response of bladder tissue from KO mice to exposure to carbachol, ATP and KCl indicates that the contractile capacity of the detrusor is not diminished by increased ECM in the bladder wall or a deleterious effect of lack of miR-29a/b1 on the muscle itself. The *in vitro* contractile response to EFS was also decreased.

Two commonly used experimental approaches, uroflowmetry and anesthetized cystometry, were performed to characterize bladder function. Uroflowmetry and cystometry are distinct assays designed to measure different urodynamic parameters that are complementary in evaluating bladder function (Ito et al., 2017; Wang et al., 2019; Wood et al., 2001). Uroflowmetry is performed in conscious, unrestrained mice, and anesthetized cystometry is done without higher cortical control of micturition (Bjorling et al., 2015; Kim and Hill, 2017). Our studies of voluntary voiding behavior in conscious mice indicated that voiding behavior in KO mice was different from that observed in WT mice, and that these animals had extended voiding duration accompanied by reduced flow rate. The bladder experiences repeated cycles of extension and recoil. The bladder wall is substantially stretched from empty to full states. The normal bladder accommodates increasing volumes of urine without a significant increase in intravesical pressure, and the mechanical properties of the bladder depend on the detrusor muscle, lamina propria, and ECM (Andersson and Arner, 2004). Decreased urinary flow rate has consistently been observed in aging rodents and humans (Kullmann et al., 2015; Osman et al., 2014), and reduced urinary flow rate and urinary retention are also hallmarks of bladder underactivity (Birder et al., 2018; Kullmann et al., 2015). Cystometry performed on anesthetized mice demonstrated that KO mice either failed to exhibit any discrete, individual micturition events or generated incomplete, irregular voids.

Decreased sensitivity of bladder innervation could arise from direct alteration of nerve structure or function. Another possible cause of this observation could be decreased conduction of current across fibrous tissue. Our findings from *in vitro* contractile studies provide mechanistic insights related to bladder dysfunction in KO mice. We found that although the intrinsic contractile properties of bladder smooth muscle cell were preserved in KO bladders, miR-29a/b1 appears necessary to maintain neurally mediated responses in WT bladders. Sadegh et al. (2012) reported that miR-29a is among the top 12 most highly expressed miRNAs in mouse bladder detrusor. Mature miRNAs are generated from cleavage of pre-miRNAs by the endonuclease Dicer (also known as Dicer1). Conditional deletion of Dicer in bladder smooth muscle cells reduced mature miR-29a abundance by 71% (Sadegh et al., 2012). Morphometric analyses revealed a significant increase in the distance between smooth muscle cells accompanied by a moderate increase of the collagen fibril size between muscle bundles. Functionally, deletion of Dicer in smooth muscle cells resulted in significant reduction in EFS-induced responses, while carbachol and ATP-induced contractions remained largely unaffected (Sadegh et al., 2012). Our results are consistent with these findings, and these observations suggest that increased ECM in the bladder wall may impair communication between muscle cells and nerves. Investigating cellular and molecular mechanisms underlying impaired neurotransmission in the presence of fibrosis of the bladder wall is an interesting area for future research. Conclusive evidence in support of a role for decreased abundance or function of potassium channels in differential *in vitro* contractile responses of bladders from KO and WT mice would require determination of expression of these channels and use of selective agonists and antagonists. Our data do not suggest a differential response to application of exogenous potassium, but the assay performed provides a summative response that is not sufficiently refined to address this question. Conclusive determination of the cause(s) of decreased response to EFS in bladder tissues obtained from the miR-29a/b1 KO mice is beyond the scope of the current study.

mRNAs encoding collagen I, III, IV and elastin were upregulated, and content of collagens and elastin proteins were increased in the bladder walls of KO mice, indicating an important role for miR-29a/b1 in maintaining bladder homeostasis and that lack of miR-29a/b1 expression is accompanied by bladder fibrosis. Message abundance and protein concentrations were measured at a single time point in the current study. The time point selected for analysis was dependent upon development of palpable bladder enlargement, and this was observed between 5 and 9 months of age in the mice we studied. The main constituents of the ECM are collagen and elastic fibers. Collagens I and III represent the bulk of the scaffolding matrix proteins in the bladder (Aitken and Bagli, 2009), and we focused our study on abundance of message and protein for these collagen isoforms. Although RNAseq did not indicate a significant increase in message for collagen 1a1 and 3a1, we did observe an increase in collagen 1a1 and 3a1 mRNA when performing RT-PCR. This is not surprising, because RNAseq is not optimally designed for measuring relative abundance of all individual mRNAs (Rachinger et al., 2021).

Collagens I and III were localized mainly in the lamina propria and surrounding smooth muscle cells. Elastin is another major ECM component, associated with collagens, permitting the ECM to recoil after stretch (Aitken and Bagli, 2009). Collagen I, III and IV, and elastin are direct targets of miR-29 (Ekman et al., 2013). In rats, spinal cord injury induced profound bladder remodeling, accompanied by significant increase in collagen and elastin content in bladder wall (Toosi et al., 2008). Similarly, streptozotocin-induced diabetes in rats was accompanied by increased bladder elastin content (Gray et al., 2008). An interesting question that remains is whether statistically different differences in abundance of message or protein content relate directly to function. As previously discussed, fibrosis of the bladder accompanies multiple disorders and is assumed to contribute significantly to impaired function.

Micturition is achieved by precisely coordinated activity of the bladder and urethra. Normal bladder emptying requires relaxation of the urethra, accompanied by increased intravesical pressure as bladder smooth muscle contracts (Andersson and Arner, 2004). We demonstrated a moderate increase in collagen content in regions of prostate immediately adjacent to the prostatic urethra. It is possible that increased collagen deposition could limit urethral relaxation during voiding, contributing to voiding dysfunction observed in KO mice. However, the relevance of observation of increased periurethral fibrosis to physiological effects remains unclear, and this remains a difficult question to directly address.

RNAseq analysis revealed hundreds of genes upregulated or downregulated in bladders of KO mice compared to those of WT mice. Lacking miR-29a/b1 expression likely initiates a chain of cellular events involving many molecules that may not be direct targets of miR-29 that may contribute to bladder remodeling and dysfunction. Among those upregulated genes, SPARC is of special interest. This secreted collagen-binding protein is a direct target of miR-29 and an important modulator of procollagen processing and collagen fibril assembly in connective tissues (Bradshaw et al., 2010; Ekman et al., 2013). The contribution of SPARC to bladder remodeling and dysfunction in KO mice clearly requires further study.

An individual miRNA can target a spectrum of mRNAs with similar or unrelated functions, providing vast regulatory potential. Increasing evidence reveals the importance of miRNAs in regulating physiological and pathophysiological processes in health and disease (Ekman et al., 2016; Harmanci et al., 2017; He et al., 2013). The role of miRNAs in regulating bladder function is an emerging research field. An intriguing finding of the current study is that lacking expression of miR-29a/b1 not only causes remarkable tissue remodeling but also impairs bladder function. Bladder fibrosis is relatively common in patients after partial bladder outlet obstruction (Metcalfe et al., 2010), neurogenic disorders (Howard et al., 2004), radiation therapy of the lower abdomen (Zwaans et al., 2020) or chronic inflammation (Grover et al., 2011), or as a natural effect of aging (Al-Zoubi et al., 2022; Lepor et al., 1992). Partial bladder outlet obstruction in patients (Gheinani et al., 2017) or rats (Ekman et al., 2013) is accompanied by bladder fibrosis and decreased miR-29.

The miR-29 family has been demonstrated to suppress translation of genes of many components of the ECM, including most isoforms of collagen as well as elastin (Ekman et al., 2016; Harmanci et al., 2017; He et al., 2013; Kriegel et al., 2012). A reduction in miR-29 has been associated with fibrosis of the liver (Matsumoto et al., 2016; Noetel et al., 2012), heart (van Rooij et al., 2008; Zhang et al., 2014), lung (Cushing et al., 2011; Xiao et al., 2012) and kidney (Liu et al., 2010; Qin et al., 2011). In dermal fibroblasts and skin tissues obtained from patients with systemic sclerosis, miR-29a expression was significantly downregulated, whereas the expression of many ECM genes was enhanced (Maurer et al., 2010; Vettori et al., 2012). Similarly, Liu et al. (2010) reported that silencing of miR-29b in the kidneys of rats caused upregulation of ECM genes including many collagen isoforms.

The effects of constitutive lack of miR-29a/b1 on other organ systems that may influence bladder function cannot be discounted. Most studies investigating organ-specific dysfunction arising in mice lack miR-29a/b1 (KO) focus on the organ system of interest and do not appear to consider how changes elsewhere may have concomitant effects on the organ system being studied. Mice that lack expression of miR-29a/b1 are smaller, have shorter life spans, and exhibit cardiac dysfunction and myocardial fibrosis, in addition to enlarged bladders (Caravia et al., 2018; van Rooij et al., 2008).

Urine retention is frequently observed in patients with LUTS, particularly those with underactive bladders (Smith, 2017). The observations from miR-29a/b1 KO mice clearly mimic some features of voiding and storage symptoms in patients with LUTS. Recent experimental studies have demonstrated that increasing tissue abundance of miR-29 using various techniques is capable of preventing or reversing fibrosis and restoring function in various murine models of fibrosis in the lung (Montgomery et al., 2014; Xiao et al., 2012), liver (Matsumoto et al., 2016), heart (Zhang et al., 2014) and kidney (Qin et al., 2011). However, a constitutive increase in miR-29 alone does not appear to be sufficient to prevent organ fibrosis associated with aging. Multiple clinical trials using miRNAs for treatment of various diseases in humans have shown promising results (Rupaimoole and Slack, 2017), and the intradermal injection of remlarsen, a synthetic miR-29b analog, for prevention of keloid formation has completed Phase II clinical trials (Gallant-Behm et al., 2019; ClinicalTrials.gov, NCT03601052). Our studies provide novel data on an important role for miR-29a in maintaining bladder function and support the therapeutic potential of miR-29a to prevent/reverse bladder fibrosis and improve symptoms in patients with LUTS and other benign bladder diseases. However, the utility of supplemental miR-29 as a therapeutic agent may be limited by a possible role for this miRNA in oncogenesis. Although miR-29 has been reported to be decreased (Wang et al., 2021; Wardana et al., 2023) or silenced by methylation (Mazzoccoli et al., 2019) concurrent to proliferation of neoplasia, all three miR-29 isoforms (a/b/c) were consistently increased in eight tumor types (Yu et al., 2017). A causal relationship has not been established between miR-29 and cancer, and the potential for miR-29 to contribute to neoplastic transformation or proliferation remains unclear; therapeutic use of miR-29 for bladder disorders should be considered in light of this.

## MATERIALS AND METHODS

### Animals

Male mice lacking expression of miR-29a and miR-29b1 (KO) and age-matched WT littermates (5-9 months old) were used. Preliminary experiments revealed significant differences between urinary dysfunction in male and female KO mice, and a decision was made to focus the current study on urinary function in male KO mice. Heterozygous breeding pairs were generously provided by Dr Adrian Liston (University of Leuven, Leuven, Belgium). These were generated using C57BL/6 mice by deleting the 10.9 kb region surrounding the miR-29a–miR-29b-1 gene that included a 1.7 kb region between the 5′ Afel site and the 3′ Sal1 site (Papadopoulou et al., 2011). Heterozygous mice backcrossed for eight generations on a C57BL/6 background were used for breeding. WT mice selected for study were age-matched littermates of KO animals. The age at which data were collected ranged from 5 to 9 months, depending upon when bladder enlargement was first detected in KO mice.

All mice were housed in Innovive^®^ microisolator cages in a room maintained on a 12 h light and dark cycle. Food and water were available *ad libitum*. Experiments were conducted in accordance with National Institutes of Health Guidelines, and all protocols were reviewed and approved by the Animal Care and Use Committee of the University of Wisconsin. Mice were weighed at the time of sacrifice, and bladder volume was also measured as previously described when mice were sacrificed (Nicholson et al., 2012; Ricke et al., 2016).

### Analysis of voluntary voiding behavior in the metabolic cages

An automated and noninvasive technique was established to evaluate voluntary voiding behavior in conscious mice (Ito et al., 2017; Wang et al., 2019; Wood et al., 2001). A major obstacle to evaluating bladder function in mice is that mice only produce a few grams of urine daily (Wood et al., 2001). Therefore, mice were brought to the testing room for acclimatization, and offered water containing 3% glucose and 0.125% saccharin to increase urine output 2 days prior to and during testing (Wang et al., 2019; Wood et al., 2001). Mice were placed individually in metabolic cages (Model 650-0322, Nalgene, Rochester, NY, USA) with floor grids optimized to minimize urine retention for 2 h between 09:00 and 11:00. A waste plate was placed on an analytical balance (New Classic MF, model MS 303S, Mettler Toledo, Columbus, OH, USA) directly under the floor grid. Video recordings of activity and patterns of waste elimination were captured using module V2 cameras (Raspberry Pi, Cambridge, UK) connected to Raspberry Pi processing units. Both Raspberry Pi units and balances were connected to a PC. Changes in weight sensed by balances were recorded, and each weight change triggered a synchronized signal to the associated Raspberry Pi unit that captured a 20 s recording of events during the corresponding weight change detected by the balance. Video recordings corresponding to each event were reviewed to verify whether the weight increase was caused by dropping of feces or urine onto the balance. The micturition pattern, defined as sustained or voiding multiple droplets, was characterized as previously described (Nicholson et al., 2012; Wang et al., 2019). Voids passing cleanly between bars of the floor grid were used to determine voided weight, voiding duration and urine flow rate. Urine flow rate was calculated based on weight of urine passed from initiation of the void to the point at which weight no longer increased (Wang et al., 2019). Mice were returned to standard housing after completion of testing.

### Cystometric evaluation of bladder function

Mice were anesthetized with urethane (1.43 g/kg, given subcutaneously). Thirty minutes later, the bladder was exposed through a lower midline abdominal incision. Bladder volume was measured with a caliper (Nicholson et al., 2012; Ricke et al., 2016), and a PE 50 cannula was inserted into the dome of the bladder and secured with a 6.0 silk purse-string suture. Muscle and skin layers were closed separately with a 5.0 silk suture as described previously (Bjorling et al., 2015; Kim and Hill, 2017). One hour after cannula implantation, the distal end of the cannula was connected to a physiological pressure transducer (Memscap AS, Skoppum, Norway) and an infusion pump (Harvard Apparatus, Holliston, MA, USA) via a three-way stopcock. Room temperature saline was infused at a rate of 0.8 ml/hour to elicit repetitive micturition. Intravesical pressure was recorded continuously using PowerLab™ instrumentation (ADInstruments, Colorado Springs, CO, USA) connected to a PC.

### Myography

Contractile function of the bladder wall was evaluated as previously described (Bartolone et al., 2020; Kullmann et al., 2014). Mice were euthanized, and bladders were rapidly collected. Bladders were cut into two strips longitudinally and placed between platinum-coated electrodes in 15 ml tissue baths containing oxygenated (95% O_2_/5% CO_2_) modified Krebs solution (pH 7.4; 137.7 mM NaCl, 1.4 mM NaH_2_PO_4_, 4.7 mM KCl, 2.5 mM CaCl_2_, 1 mM MgCl_2_, 16.3 mM NaHCO_3_ and 7.8 mM glucose) maintained at 37°C. One end of each strip was secured to a fixed metal wire with the tissue bath, and the other was attached to a force displacement transducer (Grass FT-03, Grass Instruments, West Warwick, RI, USA). Bladder strips were stretched gently to an initial tension of 9.8 mN. Tissues were allowed to equilibrate for 1 h. Concentration–response curves for CCH (0.01 to 30 μM) were constructed. In addition, responses to α,β-methylene ATP (50 μM) and KCl (60 mM) were individually determined. Responses to EFS were generated using a Grass S88 stimulator (10 V, 0.5 ms duration, pulse train 5 s, at an increasing frequency of 1-50 Hz with 3 min interval between increasing frequencies). At the end of the experiment, tissues were exposed to 137.7 mM KCl (with 0 mM NaCl) Krebs solution, and contractile responses to carbachol and EFS were normalized to KCl-induced responses in each tissue preparation (Bartolone et al., 2020; Kullmann et al., 2014).

### RNAseq analysis

Bladders were collected and stored in RNAlater (Qiagen, Valencia, CA, USA). Total RNA was extracted using Trizol reagent (Invitrogen, Carlsbad, CA, USA) and treated with DNAse I (Ambion, Austin, TX, USA) to remove genomic DNA. Preparation of sequencing libraries and Illumina sequencing were conducted at Novogene (Sacramento, CA, USA). Briefly, sequencing libraries were built using the NEBNext Ultra RNA Library Prep Kit for Illumina (NEB, Ipswich, MA, USA) and sequenced on an Illumina NovaSeq 6000 platform. Then, 150 bp paired-end reads with more than 30 million read sequencing depth were generated. The data were trimmed, quality checked and mapped to the mouse genome. Analysis of differentially expressed genes was performed using the DESeq2 R package (1.20.0). DESeq2 identifies differential expression using a model based on the negative binomial distribution. The resulting *P*-values were adjusted using the Benjamini and Hochberg's approach for controlling the false discovery rate. Genes with an adjusted *P*-value less than, or equal to, 0.05 as determined by DESeq2 were differentially expressed (Ito et al., 2021).

### Real-time PCR

A 500 ng aliquot of total RNA from each sample subsequently submitted for RNAseq analysis was used to generate first-strand cDNA using a cDNA synthesis kit following the manufacturer's instructions (Lucigen, Madison, WI, USA). Real-time PCR was performed using a CFX Connect™ Real-Time System (Bio-Rad, Hercules, CA, USA) thermocycler. Samples were amplified using the following thermal cycling conditions: 95°C for 10 min, followed by 40 cycles of amplification at 95°C for 15 s, and then 60°C for 1 min to allow for denaturing and annealing extension. Abundance of PCR product was determined semi-quantitatively using a standard curve for each gene. Expression of each gene was normalized to abundance of L19, a constitutively-expressed ribosomal protein in the same sample (Wang et al., 2008). Primer sequences used were as follows: collagen 1a1 (forward, 5′-ACGCTGCACGAGTCACAC-3′; reverse, 5′-GGCAGGCGGGAGGTCTT-3′); collagen 3a1 (forward, 5′-ACCAAAAGGTAGTGCTGGAC-3′; reverse, 5′-GACCTGGTGCTCCAGTTAGC-3′); elastin (forward, 5′-GGCGTCTTGCTGATCCTCTT-3′; reverse, 5′-ACCAGCCCCTGGATAATAGACT-3′); miR-29a precursor (forward, 5′-AGTGCACATGACCTCTTGTG-3′; reverse, 5′-TAGTCAGCTGAACGGTGCTC-3′); and L19 (forward, 5′-ATCCGCAAGCCTGTGACTGT-3′; reverse, 5′-TCGGGCCAGGGTGTTTT-3′).

### Immunohistochemistry

Bladders were decompressed to facilitate comparison between WT and KO animals. Bladders were fixed in 4% paraformaldehyde, paraffin-embedded and sectioned at 3 μm. Paraffin sections were deparaffinized in xylene and rehydrated through a series of ethanol concentrations. Sections were washed in phosphate-buffered saline (PBS), and heat-mediated antigen retrieval was achieved using Antigen Unmasking Solutions (Vector Laboratories, Newark, CA, USA; pH 9.0 for collagen I and pH 6.0 for collagen III staining) in boiling water for 20 min. Sections stained for identifying elastin were treated with 5 µg/ml proteinase K for 5 min at room temperature. Tissues were rinsed in PBS and blocked with 10% normal donkey serum for 1 h at room temperature, and the primary antibody was applied and incubated for 48 h at 4°C. Tissues were then rinsed in PBS and incubated with an Alexa Fluor^®^ 488-conjugated donkey anti-rabbit secondary antibody (Life Technologies Carlsbad, CA, USA) for 90 min at room temperature. Tissues were washed in PBS and mounted with Vectashield Plus antifading solution containing 4′,6-diamidino-2-phenylindole (DAPI; Vector Laboratories). Images were acquired with a 600 Nikon Eclipse microscopy and a QICAM CCD camera (QImaging, Surrey, Canada), interfaced to NIS elements imaging software (Nikon Instruments Inc., Melville, NY, USA). The primary antibodies used were rabbit anti-collagen I (ab254113; 1:100 dilution) (Hao et al., 2021), rabbit anti-collagen III (ab278080; 1:3000 dilution) (Rusu et al., 2019) and rabbit anti-elastin (ab21610; 1:100 dilution) (Wegner et al., 2020), obtained from Abcam (Waltham, MA, USA). Antibody specificity was confirmed by the companies that supplied them and by the citations referenced.

### Measurement of collagen and elastin contents in the detrusor

After myograph experiments, collagen and elastin content within the detrusor was determined. Mucosa was carefully removed, and detrusor was weighed. Then, 3 mg of detrusor was collected, and collagen was measured using Sircol™ Collagen Assay from Biocolor (Carrickfergus, UK) following the manufacturer's instructions. Briefly, tissue was minced using a fine scissor and digested with 0.1 mg/ml pepsin overnight at 4°C. This solution was centrifuged at 12,000 ***g*** for 10 min, and supernatant was carefully transferred to a clean tube and treated with an acid-neutralizing regent included in the assay kit. The collagen content was then determined using the Sircol Dye Reagent (Gray et al., 2008; Toosi et al., 2008).

Similarly, elastin was measured using Fastin™ Elastin Assay (Biocolor) following the manufacturer's instructions. Elastin was extracted with 0.25 M oxalic acid at 100°C for 2 h and then precipitated using an Elastin Precipitating Reagent included in the assay kit. The elastin content was measured using the Elastin Dye Reagent (Gray et al., 2008; Toosi et al., 2008).

Both collagen and elastin content were normalized to the wet tissue weight for each sample.

### Measurement of collagen abundance in prostatic urethra

The mouse prostate is far more complex than the human prostate, and comprises multiple lobes that extend away from their connection to the urethra. It has previously been observed that the periurethral prostatic tissue may more closely resemble that of humans, and the prostatic urethra has been recognized as a ‘bottleneck’ in the male urethra. Therefore, we determined collagen abundance in prostatic tissue immediately adjacent to the urethra (Macoska et al., 2019).

Tissues were paraffin embedded, sectioned, deparaffinized and rehydrated. At least three sections containing prostatic urethra, ejaculatory ducts and seminiferous tubules were selected for analysis. Collagens were stained with PSR (60941, Abcam, Cambridge, UK). PSR is a strong anionic dye that binds to cationic collagen fibers, and PSR staining to identify fibrillar collagen has been widely used as a sensitive and specific stain for collagen in histological sections (Macoska et al., 2019). Sections were incubated for 90 min with PSR solution, following the manufacturer's instructions. Slides were rinsed with acidified water (0.5% acetic acid), dehydrated with graded ethanol, cleared in xylene and cover slipped with Richard-Allan toluene-based mounting medium (Thermo Fisher Scientific, Waltham, MA, USA). PSR-stained sections yield a strong red fluorescence signal that is specific for collagens (Wegner et al., 2017). Slides were viewed with a Nikon Eclipse E600 microscope (Nikon Instruments Inc.), and fluorescent images were digitally captured with a QICAM CCD camera (Teledyne Princeton Instruments, Trenton, NJ, USA). Tissue adjacent to the urethra routinely showed the most intense PSR staining, and this area was identified as the region of interest for quantification of collagen abundance using ImageJ (National Institutes of Health, Bethesda, MD, USA). Pixels associated with PSR staining were reported as a percentage of total pixels within the region of interest (Macoska et al., 2019; Wegner et al., 2017).

### Statistical analysis

Data were found to be normally distributed, and an unpaired two-tailed Student's *t*-test was used for comparison between groups. *P*<0.05 was considered significant. Data are shown as box and whiskers plots in which the center line indicates the median of the dataset, box limits indicate upper and lower quartiles, and whiskers show data minimum and maximum.

## Supplementary Material

10.1242/dmm.050054_sup1 Supplementary information

## References

[DMM050054C1] Aitken, K. J. and Bagli, D. J. (2009). The bladder extracellular matrix. Part I: architecture, development and disease. *Nat. Rev. Urol.* 6, 596-611. 10.1038/nrurol.2009.201 19890339

[DMM050054C2] Al-Zoubi, R. M., Alwani, M., Aboumarzouk, O. M., Elaarag, M., Al-Qudimat, A. R., Ojha, L. and Yassin, A. (2022). Updates on androgen replacement therapy and lower urinary tract symptoms: a narrative review. *Aging Male* 25, 234-241. 10.1080/13685538.2022.2118253 36066424

[DMM050054C3] Andersson, K. E. (2003). Storage and voiding symptoms: pathophysiologic aspects. *Urology* 62, 3-10. 10.1016/j.urology.2003.09.030 14662401

[DMM050054C4] Andersson, K. E. and Arner, A. (2004). Urinary bladder contraction and relaxation: physiology and pathophysiology. *Physiol. Rev.* 84, 935-986. 10.1152/physrev.00038.2003 15269341

[DMM050054C5] Bartolone, S. N., Ward, E. P., Wang, Z., Zwaans, B. M., Chancellor, M. B., Bjorling, D. E. and Lamb, L. E. (2020). Micturition defects and altered bladder function in the klotho mutant mouse model of aging. *Am. J. Clin. Exp. Urol.* 8, 81-92. 32699807 PMC7364365

[DMM050054C6] Birder, L. A., Kullmann, A. F. and Chapple, C. R. (2018). The aging bladder insights from animal models. *Asian J. Urol.* 5, 135-140. 10.1016/j.ajur.2017.03.004 29988876 PMC6033201

[DMM050054C7] Bjorling, D. E., Wang, Z., Vezina, C. M., Ricke, W. A., Keil, K. P., Yu, W., Guo, L., Zeidel, M. L. and Hill, W. G. (2015). Evaluation of voiding assays in mice: impact of genetic strains and sex. *Am. J. Physiol. Renal. Physiol.* 308, F1369-F1378. 10.1152/ajprenal.00072.2015 25904700 PMC4469884

[DMM050054C8] Bradshaw, A. D., Baicu, C. F., Rentz, T. J., Van Laer, A. O., Bonnema, D. D. and Zile, M. R. (2010). Age-dependent alterations in fibrillar collagen content and myocardial diastolic function: role of SPARC in post-synthetic procollagen processing. *Am. J. Physiol. Heart Circ. Physiol.* 298, H614-H622. 10.1152/ajpheart.00474.2009 20008277 PMC2822576

[DMM050054C9] Caravia, X. M., Fanjul, V., Oliver, E., Roiz-Valle, D., Moran-Alvarez, A., Desdin-Mico, G., Mittelbrunn, M., Cabo, R., Vega, J. A., Rodriguez, F. et al. (2018). The microRNA-29/PGC1alpha regulatory axis is critical for metabolic control of cardiac function. *PLoS Biol.* 16, e2006247. 10.1371/journal.pbio.2006247 30346946 PMC6211751

[DMM050054C10] Chang, T. C., Yu, D., Lee, Y. S., Wentzel, E. A., Arking, D. E., West, K. M., Dang, C. V., Thomas-Tikhonenko, A. and Mendell, J. T. (2008). Widespread microRNA repression by Myc contributes to tumorigenesis. *Nat. Genet.* 40, 43-50. 10.1038/ng.2007.30 18066065 PMC2628762

[DMM050054C11] Chapple, C. R., Wein, A. J., Abrams, P., Dmochowski, R. R., Giuliano, F., Kaplan, S. A., McVary, K. T. and Roehrborn, C. G. (2008). Lower urinary tract symptoms revisited: a broader clinical perspective. *Eur. Urol.* 54, 563-569. 10.1016/j.eururo.2008.03.109 18423969

[DMM050054C12] Cushing, L., Kuang, P. P., Qian, J., Shao, F., Wu, J., Little, F., Thannickal, V. J., Cardoso, W. V. and Lu, J. (2011). miR-29 is a major regulator of genes associated with pulmonary fibrosis. *Am. J. Respir. Cell Mol. Biol.* 45, 287-294. 10.1165/rcmb.2010-0323OC 20971881 PMC3175558

[DMM050054C13] Deveaud, C. M., Macarak, E. J., Kucich, U., Ewalt, D. H., Abrams, W. R. and Howard, P. S. (1998). Molecular analysis of collagens in bladder fibrosis. *J. Urol.* 160, 1518-1527. 10.1016/S0022-5347(01)62606-5 9751406

[DMM050054C14] Edgar, R., Domrachev, M. and Lash, A. E. (2002). Gene Expression Omnibus: NCBI gene expression and hybridization array data repository. *Nucleic Acids Res.* 30, 207-210. 10.1093/nar/30.1.207 11752295 PMC99122

[DMM050054C15] Ekman, M., Albinsson, S., Uvelius, B. and Sward, K. (2016). MicroRNAs in bladder outlet obstruction: relationship to growth and matrix remodelling. *Basic Clin. Pharmacol. Toxicol.* 119 Suppl. 3, 5-17. 10.1111/bcpt.12534 26612603

[DMM050054C16] Ekman, M., Bhattachariya, A., Dahan, D., Uvelius, B., Albinsson, S. and Sward, K. (2013). Mir-29 repression in bladder outlet obstruction contributes to matrix remodeling and altered stiffness. *PLoS ONE* 8, e82308. 10.1371/journal.pone.0082308 24340017 PMC3858279

[DMM050054C17] Friedman, R. C., Farh, K. K., Burge, C. B. and Bartel, D. P. (2009). Most mammalian mRNAs are conserved targets of microRNAs. *Genome Res.* 19, 92-105. 10.1101/gr.082701.108 18955434 PMC2612969

[DMM050054C18] Fry, C. H., Kitney, D. G., Paniker, J., Drake, M. J., Kanai, A. and Andersson, K. E. (2018). Fibrosis and the bladder, implications for function ICI-RS 2017. *Neurourol. Urodyn.* 37, S7-s12. 10.1002/nau.23725 30133788

[DMM050054C19] Gallant-Behm, C. L., Piper, J., Lynch, J. M., Seto, A. G., Hong, S. J., Mustoe, T. A., Maari, C., Pestano, L. A., Dalby, C. M., Jackson, A. L. et al. (2019). A MicroRNA-29 mimic (Remlarsen) represses extracellular matrix expression and fibroplasia in the skin. *J. Invest. Dermatol.* 139, 1073-1081. 10.1016/j.jid.2018.11.007 30472058

[DMM050054C20] Ge, M., Liu, C., Li, L., Lan, M., Yu, Y., Gu, L., Su, Y., Zhang, K., Zhang, Y., Wang, T. et al. (2019). miR-29a/b1 inhibits hair follicle stem cell lineage progression by spatiotemporally suppressing WNT and BMP signaling. *Cell Rep.* 29, 2489-2504.e2484. 10.1016/j.celrep.2019.10.062 31747615

[DMM050054C21] Gheinani, A. H., Kiss, B., Moltzahn, F., Keller, I., Bruggmann, R., Rehrauer, H., Fournier, C. A., Burkhard, F. C. and Monastyrskaya, K. (2017). Characterization of miRNA-regulated networks, hubs of signaling, and biomarkers in obstruction-induced bladder dysfunction. *JCI Insight* 2, e89560. 10.1172/jci.insight.89560 28138557 PMC5256140

[DMM050054C22] Gray, M. A., Wang, C. C., Sacks, M. S., Yoshimura, N., Chancellor, M. B. and Nagatomi, J. (2008). Time-dependent alterations of select genes in streptozotocin-induced diabetic rat bladder. *Urology* 71, 1214-1219. 10.1016/j.urology.2007.11.054 18279932

[DMM050054C23] Grover, S., Srivastava, A., Lee, R., Tewari, A. K. and Te, A. E. (2011). Role of inflammation in bladder function and interstitial cystitis. *Ther. Adv. Urol.* 3, 19-33. 10.1177/1756287211398255 21789096 PMC3126088

[DMM050054C24] Hao, Y., Miao, J., Liu, W., Cai, K., Huang, X. and Peng, L. (2021). Mesenchymal stem cell-derived exosomes carry MicroRNA-125a to protect against diabetic nephropathy by targeting histone deacetylase 1 and downregulating endothelin-1. *Diabetes Metab. Syndr. Obes.* 14, 1405-1418. 10.2147/DMSO.S286191 33790607 PMC8006976

[DMM050054C25] Harmanci, D., Erkan, E. P., Kocak, A. and Akdogan, G. G. (2017). Role of the microRNA-29 family in fibrotic skin diseases. *Biomed Rep* 6, 599-604. 10.3892/br.2017.900 28584629 PMC5449962

[DMM050054C26] He, Y., Huang, C., Lin, X. and Li, J. (2013). MicroRNA-29 family, a crucial therapeutic target for fibrosis diseases. *Biochimie* 95, 1355-1359. 10.1016/j.biochi.2013.03.010 23542596

[DMM050054C27] Howard, P. S., Renfrow, D., Schechter, N. M. and Kucich, U. (2004). Mast cell chymase is a possible mediator of neurogenic bladder fibrosis. *Neurourol. Urodyn.* 23, 374-382. 10.1002/nau.20032 15227657

[DMM050054C28] Huang, D. W., Sherman, B. T., Tan, Q., Collins, J. R., Alvord, W. G., Roayaei, J., Stephens, R., Baseler, M. W., Lane, H. C. and Lempicki, R. A. (2007). The DAVID Gene Functional Classification Tool: a novel biological module-centric algorithm to functionally analyze large gene lists. *Genome Biol.* 8, R183. 10.1186/gb-2007-8-9-r183 17784955 PMC2375021

[DMM050054C29] Ito, H., Pickering, A. E., Igawa, Y., Kanai, A. J., Fry, C. H. and Drake, M. J. (2017). Muro-neuro-urodynamics; a review of the functional assessment of mouse lower urinary tract function. *Front. Physiol.* 8, 49. 10.3389/fphys.2017.00049 28220079 PMC5292568

[DMM050054C30] Ito, S., Nomura, T., Ueda, T., Inui, S., Morioka, Y., Honjo, H., Fukui, A., Fujihara, A., Hongo, F. and Ukimura, O. (2021). Gene expression profiles during tissue remodeling following bladder outlet obstruction. *Sci. Rep.* 11, 13171. 10.1038/s41598-021-92756-1 34162983 PMC8222387

[DMM050054C31] Kamei, J., Ito, H., Aizawa, N., Hotta, H., Kojima, T., Fujita, Y., Ito, M., Homma, Y. and Igawa, Y. (2018). Age-related changes in function and gene expression of the male and female mouse bladder. *Sci. Rep.* 8, 2089. 10.1038/s41598-018-20406-0 29391518 PMC5794976

[DMM050054C32] Kanehisa, M., Furumichi, M., Sato, Y., Kawashima, M. and Ishiguro-Watanabe, M. (2023). KEGG for taxonomy-based analysis of pathways and genomes. *Nucleic Acids Res.* 51, D587-d592. 10.1093/nar/gkac963 36300620 PMC9825424

[DMM050054C33] Kim, A. K. and Hill, W. G. (2017). Effect of filling rate on cystometric parameters in young and middle aged mice. *Bladder* 4, e28. 28553656 10.14440/bladder.2017.88 PMC5443651

[DMM050054C34] Kim, J. H., Lee, B. R., Choi, E. S., Lee, K. M., Choi, S. K., Cho, J. H., Jeon, W. B. and Kim, E. (2017). Reverse expression of aging-associated molecules through transfection of miRNAs to aged mice. *Mol. Ther. Nucleic Acids* 6, 106-115. 10.1016/j.omtn.2016.11.005 28325277 PMC5363412

[DMM050054C35] Kriegel, A. J., Liu, Y., Fang, Y., Ding, X. and Liang, M. (2012). The miR-29 family: genomics, cell biology, and relevance to renal and cardiovascular injury. *Physiol. Genomics* 44, 237-244. 10.1152/physiolgenomics.00141.2011 22214600 PMC3289120

[DMM050054C36] Kullmann, F. A., Birder, L. A. and Andersson, K. E. (2015). Translational research and functional changes in voiding function in older adults. *Clin. Geriatr. Med.* 31, 535-548. 10.1016/j.cger.2015.06.001 26476114 PMC4865381

[DMM050054C37] Kullmann, F. A., Daugherty, S. L., de Groat, W. C. and Birder, L. A. (2014). Bladder smooth muscle strip contractility as a method to evaluate lower urinary tract pharmacology. *J. Vis. Exp.* e51807. 10.3791/51807 25178111 PMC4435542

[DMM050054C38] Lai, E. C. (2002). Micro RNAs are complementary to 3’ UTR sequence motifs that mediate negative post-transcriptional regulation. *Nat. Genet.* 30, 363-364. 10.1038/ng865 11896390

[DMM050054C39] Lepor, H., Sunaryadi, I., Hartanto, V. and Shapiro, E. (1992). Quantitative morphometry of the adult human bladder. *J. Urol.* 148, 414-417. 10.1016/S0022-5347(17)36619-3 1378909

[DMM050054C40] Lewis, B. P., Burge, C. B. and Bartel, D. P. (2005). Conserved seed pairing, often flanked by adenosines, indicates that thousands of human genes are microRNA targets. *Cell* 120, 15-20. 10.1016/j.cell.2004.12.035 15652477

[DMM050054C41] Lim, L. P., Lau, N. C., Garrett-Engele, P., Grimson, A., Schelter, J. M., Castle, J., Bartel, D. P., Linsley, P. S. and Johnson, J. M. (2005). Microarray analysis shows that some microRNAs downregulate large numbers of target mRNAs. *Nature* 433, 769-773. 10.1038/nature03315 15685193

[DMM050054C42] Liu, Y., Taylor, N. E., Lu, L., Usa, K., Cowley, A. W., Jr, Ferreri, N. R., Yeo, N. C. and Liang, M. (2010). Renal medullary microRNAs in Dahl salt-sensitive rats: miR-29b regulates several collagens and related genes. *Hypertension* 55, 974-982. 10.1161/HYPERTENSIONAHA.109.144428 20194304 PMC2862728

[DMM050054C43] Ma, Y., Murgia, N., Liu, Y., Li, Z., Sirakawin, C., Konovalov, R., Kovzel, N., Xu, Y., Kang, X., Tiwari, A. et al. (2022). Neuronal miR-29a protects from obesity in adult mice. *Mol. Metab.* 61, 101507. 10.1016/j.molmet.2022.101507 35490865 PMC9114687

[DMM050054C44] Macoska, J. A., Wang, Z., Virta, J., Zacharias, N. and Bjorling, D. E. (2019). Inhibition of the CXCL12/CXCR4 axis prevents periurethral collagen accumulation and lower urinary tract dysfunction in vivo. *Prostate* 79, 757-767. 10.1002/pros.23781 30811623 PMC7269149

[DMM050054C45] Matsumoto, Y., Itami, S., Kuroda, M., Yoshizato, K., Kawada, N. and Murakami, Y. (2016). MiR-29a Assists in Preventing the Activation of Human Stellate Cells and Promotes Recovery From Liver Fibrosis in Mice. *Mol. Ther.* 24, 1848-1859. 10.1038/mt.2016.127 27480597 PMC5112039

[DMM050054C46] Maurer, B., Stanczyk, J., Jungel, A., Akhmetshina, A., Trenkmann, M., Brock, M., Kowal-Bielecka, O., Gay, R. E., Michel, B. A., Distler, J. H. et al. (2010). MicroRNA-29, a key regulator of collagen expression in systemic sclerosis. *Arthritis. Rheum.* 62, 1733-1743. 10.1002/art.27443 20201077

[DMM050054C47] Mazzoccoli, L., Robaina, M. C., Bacchi, C. E., Soares Lima, S. C. and Klumb, C. E. (2019). miR-29 promoter and enhancer methylation identified by pyrosequencing in Burkitt lymhoma cells: Interplay between MYC and miR-29 regulation. *Oncol. Rep.* 42, 775-784. 10.3892/or.2019.7183 31173259

[DMM050054C83] Metcalfe, P. D., Wang, J., Jiao, H., Huang, Y., Hori, K., Moore, R. B. and Tredget, E. E. (2010). Bladder outlet obstruction: progression from inflammation to fibrosis. *BJU Int.* 106, 1686-1694. 10.1111/j.1464-410X.2010.09445.x 20590549

[DMM050054C48] Mirone, V., Imbimbo, C., Sessa, G., Palmieri, A., Longo, N., Granata, A. M. and Fusco, F. (2004). Correlation between detrusor collagen content and urinary symptoms in patients with prostatic obstruction. *J. Urol.* 172, 1386-1389. 10.1097/01.ju.0000139986.08972.e3 15371851

[DMM050054C49] Montgomery, R. L., Yu, G., Latimer, P. A., Stack, C., Robinson, K., Dalby, C. M., Kaminski, N. and van Rooij, E. (2014). MicroRNA mimicry blocks pulmonary fibrosis. *EMBO Mol. Med.* 6, 1347-1356. 10.15252/emmm.201303604 25239947 PMC4287936

[DMM050054C50] Mott, J. L., Kurita, S., Cazanave, S. C., Bronk, S. F., Werneburg, N. W. and Fernandez-Zapico, M. E. (2010). Transcriptional suppression of mir-29b-1/mir-29a promoter by c-Myc, hedgehog, and NF-kappaB. *J. Cell. Biochem.* 110, 1155-1164. 10.1002/jcb.22630 20564213 PMC2922950

[DMM050054C51] Nicholson, T. M., Ricke, E. A., Marker, P. C., Miano, J. M., Mayer, R. D., Timms, B. G., vom Saal, F. S., Wood, R. W. and Ricke, W. A. (2012). Testosterone and 17beta-estradiol induce glandular prostatic growth, bladder outlet obstruction, and voiding dysfunction in male mice. *Endocrinology* 153, 5556-5565. 10.1210/en.2012-1522 22948219 PMC3473198

[DMM050054C52] Noetel, A., Kwiecinski, M., Elfimova, N., Huang, J. and Odenthal, M. (2012). microRNA are Central Players in Anti- and Profibrotic Gene Regulation during Liver Fibrosis. *Front. Physiol.* 3, 49. 10.3389/fphys.2012.00049 22457651 PMC3307137

[DMM050054C53] O'Reilly, S. (2016). MicroRNAs in fibrosis: opportunities and challenges. *Arthritis Res. Ther.* 18, 11. 10.1186/s13075-016-0929-x 26762516 PMC4718015

[DMM050054C54] Osman, N. I., Chapple, C. R., Abrams, P., Dmochowski, R., Haab, F., Nitti, V., Koelbl, H., van Kerrebroeck, P. and Wein, A. J. (2014). Detrusor underactivity and the underactive bladder: a new clinical entity? A review of current terminology, definitions, epidemiology, aetiology, and diagnosis. *Eur. Urol.* 65, 389-398. 10.1016/j.eururo.2013.10.015 24184024

[DMM050054C55] Papadopoulou, A. S., Dooley, J., Linterman, M. A., Pierson, W., Ucar, O., Kyewski, B., Zuklys, S., Hollander, G. A., Matthys, P., Gray, D. H. et al. (2011). The thymic epithelial microRNA network elevates the threshold for infection-associated thymic involution via miR-29a mediated suppression of the IFN-alpha receptor. *Nat. Immunol.* 13, 181-187. 10.1038/ni.2193 22179202 PMC3647613

[DMM050054C56] Paul, P., Chakraborty, A., Sarkar, D., Langthasa, M., Rahman, M., Bari, M., Singha, R. S., Malakar, A. K. and Chakraborty, S. (2018). Interplay between miRNAs and human diseases. *J. Cell. Physiol.* 233, 2007-2018. 10.1002/jcp.25854 28181241

[DMM050054C57] Qin, W., Chung, A. C., Huang, X. R., Meng, X. M., Hui, D. S., Yu, C. M., Sung, J. J. and Lan, H. Y. (2011). TGF-beta/Smad3 signaling promotes renal fibrosis by inhibiting miR-29. *J. Am. Soc. Nephrol.* 22, 1462-1474. 10.1681/ASN.2010121308 21784902 PMC3148701

[DMM050054C58] Rachinger, N., Fischer, S., Böhme, I., Linck-Paulus, L., Kuphal, S., Kappelmann-Fenzl, M. and Bosserhoff, A. K. (2021). Loss of gene information: discrepancies between RNA sequencing, cDNA microarray, and qRT-PCR. *Int. J. Mol. Sci.* 22, 9349. 10.3390/ijms22179349 34502254 PMC8430810

[DMM050054C59] Ricke, W. A., Lee, C. W., Clapper, T. R., Schneider, A. J., Moore, R. W., Keil, K. P., Abler, L. L., Wynder, J. L., López Alvarado, A., Beaubrun, I. et al. (2016). In utero and lactational TCDD exposure increases susceptibility to lower urinary tract dysfunction in adulthood. *Toxicol. Sci.* 150, 429-440. 10.1093/toxsci/kfw009 26865671 PMC4900134

[DMM050054C60] Rupaimoole, R. and Slack, F. J. (2017). MicroRNA therapeutics: towards a new era for the management of cancer and other diseases. *Nat. Rev. Drug Discov.* 16, 203-222. 10.1038/nrd.2016.246 28209991

[DMM050054C61] Rusu, M., Hilse, K., Schuh, A., Martin, L., Slabu, I., Stoppe, C. and Liehn, E. A. (2019). Biomechanical assessment of remote and postinfarction scar remodeling following myocardial infarction. *Sci. Rep.* 9, 16744. 10.1038/s41598-019-53351-7 31727993 PMC6856121

[DMM050054C62] Sadegh, M. K., Ekman, M., Rippe, C., Uvelius, B., Sward, K. and Albinsson, S. (2012). Deletion of Dicer in smooth muscle affects voiding pattern and reduces detrusor contractility and neuroeffector transmission. *PLoS ONE* 7, e35882. 10.1371/journal.pone.0035882 22558254 PMC3338793

[DMM050054C63] Salomon, W. E., Jolly, S. M., Moore, M. J., Zamore, P. D. and Serebrov, V. (2015). Single-molecule imaging reveals that argonaute reshapes the binding properties of its nucleic acid guides. *Cell* 162, 84-95. 10.1016/j.cell.2015.06.029 26140592 PMC4503223

[DMM050054C64] Schirle, N. T., Sheu-Gruttadauria, J. and MacRae, I. J. (2014). Structural basis for microRNA targeting. *Science* 346, 608-613. 10.1126/science.1258040 25359968 PMC4313529

[DMM050054C65] Smith, P. P. (2017). Pathophysiology of the underactive bladder: evolving new concepts. *Curr. Bladder Dysfunct. Rep.* 12, 35-41. 10.1007/s11884-017-0407-6 28740567 PMC5521014

[DMM050054C66] Thomas, A. W. and Abrams, P. (2000). Lower urinary tract symptoms, benign prostatic obstruction and the overactive bladder. *BJU Int.* 85 Suppl 3, 57-68; discussion 70-51. 10.1111/j.1464-410X.2000.tb16953.x 11954200

[DMM050054C67] Toosi, K. K., Nagatomi, J., Chancellor, M. B. and Sacks, M. S. (2008). The effects of long-term spinal cord injury on mechanical properties of the rat urinary bladder. *Ann. Biomed. Eng.* 36, 1470-1480. 10.1007/s10439-008-9525-9 18622703

[DMM050054C68] van Rooij, E., Sutherland, L. B., Thatcher, J. E., DiMaio, J. M., Naseem, R. H., Marshall, W. S., Hill, J. A. and Olson, E. N. (2008). Dysregulation of microRNAs after myocardial infarction reveals a role of miR-29 in cardiac fibrosis. *Proc. Natl. Acad. Sci. USA* 105, 13027-13032. 10.1073/pnas.0805038105 18723672 PMC2529064

[DMM050054C69] Vasquez, E., Cristofaro, V., Lukianov, S., Burkhard, F. C., Gheinani, A. H., Monastyrskaya, K., Bielenberg, D. R., Sullivan, M. P. and Adam, R. M. (2017). Deletion of neuropilin 2 enhances detrusor contractility following bladder outlet obstruction. *JCI Insight* 2, e90617. 10.1172/jci.insight.90617 28194441 PMC5291730

[DMM050054C70] Vettori, S., Gay, S. and Distler, O. (2012). Role of MicroRNAs in fibrosis. *Open Rheumatol. J.* 6, 130-139. 10.2174/1874312901206010130 22802911 PMC3396185

[DMM050054C71] Wang, L., Guan, X., Hu, Q., Wu, Z., Chen, W., Song, L., Wang, K., Tian, K., Cao, C., Zhang, D. et al. (2021). TGFB3 downregulation causing chordomagenesis and its tumor suppression role maintained by Smad7. *Carcinogenesis* 42, 913-923. 10.1093/carcin/bgab022 34057989

[DMM050054C72] Wang, Z., Guzman, E. C., Nimunkar, A., Keil, K. P., Vezina, C. M., Ricke, W. A., Macoska, J. and Bjorling, D. E. (2019). Void sorcerer: an open source, open access framework for mouse uroflowmetry. *Am. J. Clin. Exp. Urol.* 7, 170-177. 31317056 PMC6627548

[DMM050054C73] Wang, Z. Y., Wang, P., Merriam, F. V. and Bjorling, D. E. (2008). Lack of TRPV1 inhibits cystitis-induced increased mechanical sensitivity in mice. *Pain* 139, 158-167. 10.1016/j.pain.2008.03.020 18445509

[DMM050054C74] Wardana, T., Oktriani, R., Murjayanto, C. H., Putri, D. U., Anwar, S. L., Aryandono, T. and Haryana, S. M. (2023). MicroRNA gene signature for predicting mechanisms in nasopharyngeal carcinoma: a case study on the potential application of circulating biomarkers. *Microrna* 12, 29-44. 10.2174/2211536611666220919144834 36121076

[DMM050054C75] Wegner, K. A., Keikhosravi, A., Eliceiri, K. W. and Vezina, C. M. (2017). Fluorescence of picrosirius red multiplexed with immunohistochemistry for the quantitative assessment of collagen in tissue sections. *J. Histochem. Cytochem.* 65, 479-490. 10.1369/0022155417718541 28692327 PMC5533271

[DMM050054C76] Wegner, K. A., Mueller, B. R., Unterberger, C. J., Avila, E. J., Ruetten, H., Turco, A. E., Oakes, S. R., Girardi, N. M., Halberg, R. B., Swanson, S. M. et al. (2020). Prostate epithelial-specific expression of activated PI3K drives stromal collagen production and accumulation. *J. Pathol.* 250, 231-242. 10.1002/path.5363 31674011 PMC7071816

[DMM050054C77] Wood, R., Eichel, L., Messing, E. M. and Schwarz, E. (2001). Automated noninvasive measurement of cyclophosphamide-induced changes in murine voiding frequency and volume. *J. Urol.* 165, 653-659. 10.1097/00005392-200102000-00089 11176453

[DMM050054C78] Xiao, J., Meng, X. M., Huang, X. R., Chung, A. C., Feng, Y. L., Hui, D. S., Yu, C. M., Sung, J. J. and Lan, H. Y. (2012). miR-29 inhibits bleomycin-induced pulmonary fibrosis in mice. *Mol. Ther.* 20, 1251-1260. 10.1038/mt.2012.36 22395530 PMC3369297

[DMM050054C79] Yu, W., Hill, W. G., Robson, S. C. and Zeidel, M. L. (2018). Role of P2X4 receptor in mouse voiding function. *Sci. Rep.* 8, 1838. 10.1038/s41598-018-20216-4 29382907 PMC5789870

[DMM050054C80] Yu, W., Zhao, S., Wang, Y., Zhao, B. N., Zhao, W. and Zhou, X. (2017). Identification of cancer prognosis-associated functional modules using differential co-expression networks. *Oncotarget* 8, 112928-112941. 10.18632/oncotarget.22878 29348878 PMC5762563

[DMM050054C81] Zhang, Y., Huang, X. R., Wei, L. H., Chung, A. C., Yu, C. M. and Lan, H. Y. (2014). miR-29b as a therapeutic agent for angiotensin II-induced cardiac fibrosis by targeting TGF-beta/Smad3 signaling. *Mol. Ther.* 22, 974-985. 10.1038/mt.2014.25 24569834 PMC4015231

[DMM050054C82] Zwaans, B. M. M., Nicolai, H. E., Chancellor, M. B. and Lamb, L. E. (2020). Prostate cancer survivors with symptoms of radiation cystitis have elevated fibrotic and vascular proteins in urine. *PLoS ONE* 15, e0241388. 33119677 10.1371/journal.pone.0241388 PMC7595289

