# Supplementary material for: Lack of expression of miR-29a/b1 impairs bladder function in male mice
Source: Dis Model Mech. 2023 Jun 7;16(6):dmm050054. doi: 10.1242/dmm.050054 (PMC10259841; doi:10.1242/dmm.050054)
Supplement: Supplementary information [file dmm-16-050054-s1.pdf]

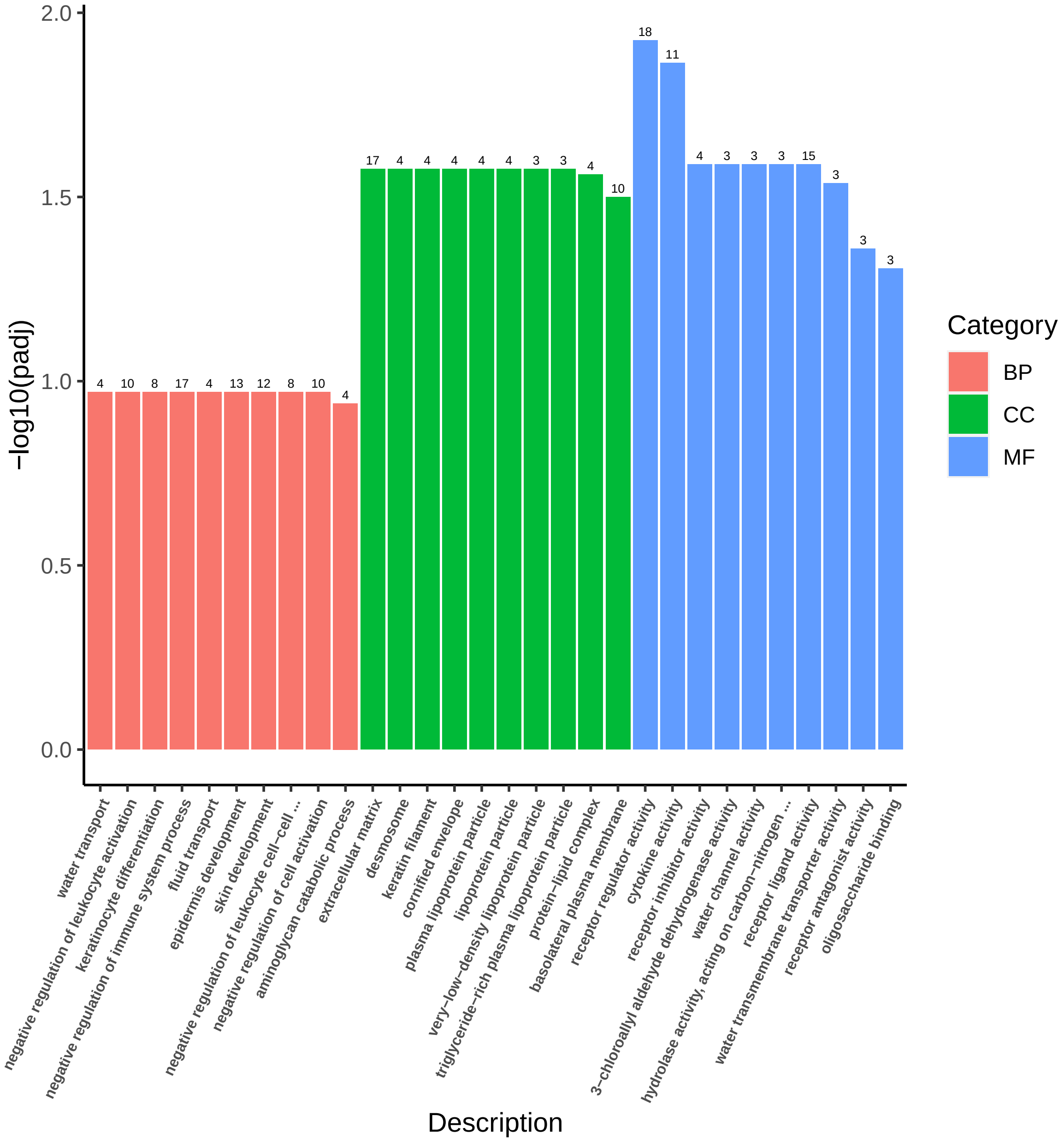

Fig. S1. Results of investigation of gene ontology for those signals found to be upregulated in bladders from KO mice relative to those from WT mice. BP - biological process; CC - cellular component; MF -molecular function.

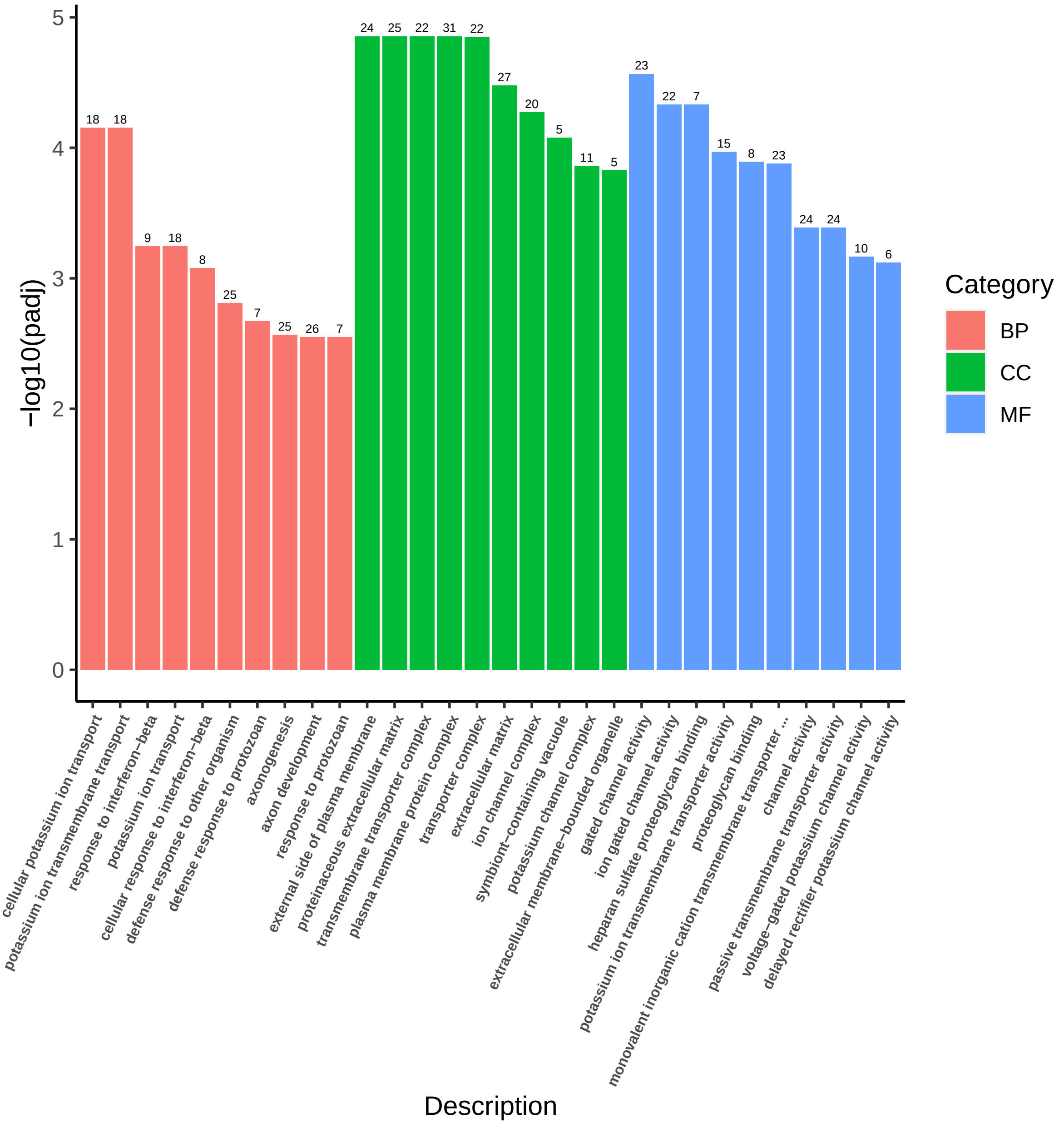

Fig. S2. Results of investigation of gene ontology for those signals found to be downregulated in bladders from KO mice relative to those from WT mice. BP - biological process; CC - cellular component; MF -molecular function.

Table S1. All genes found to be differentially expressed by bladders of KO mice compared to those of WT mice.

Click here to download Table S1

Disease Models & Mechanisms • Supplementary information

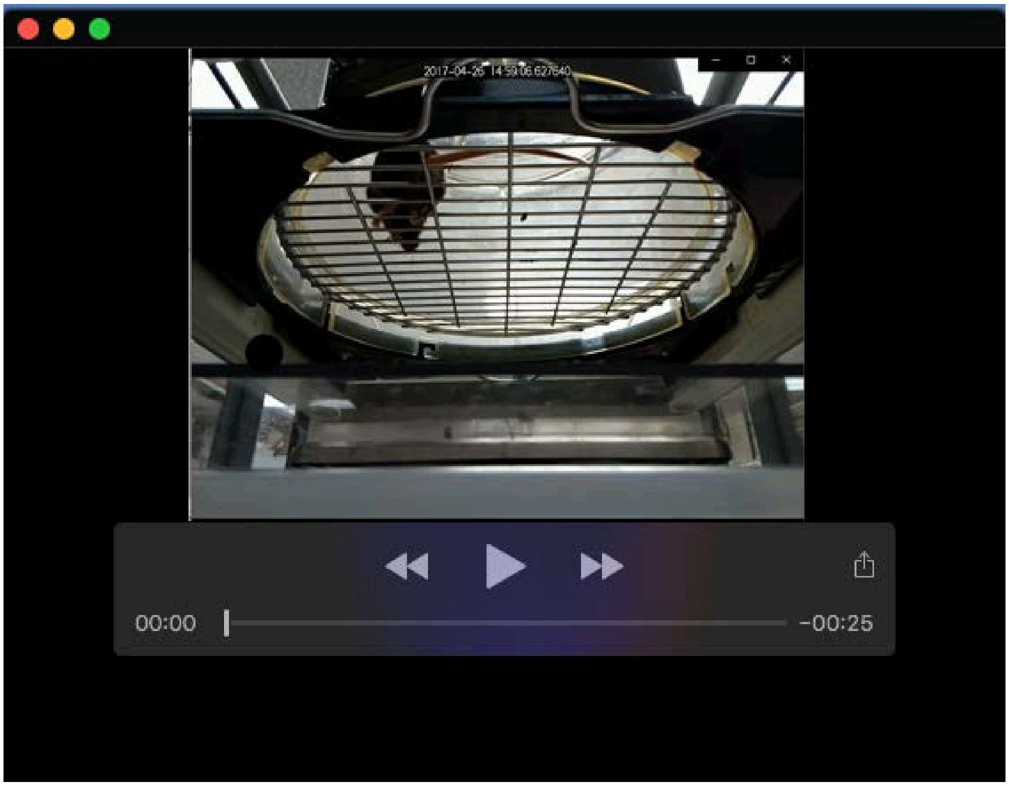

Movie 1. Voiding pattern of WT mouse. This video illustrates the voiding pattern characteristic of male wild-type mice that voided a continuous stream of urine.

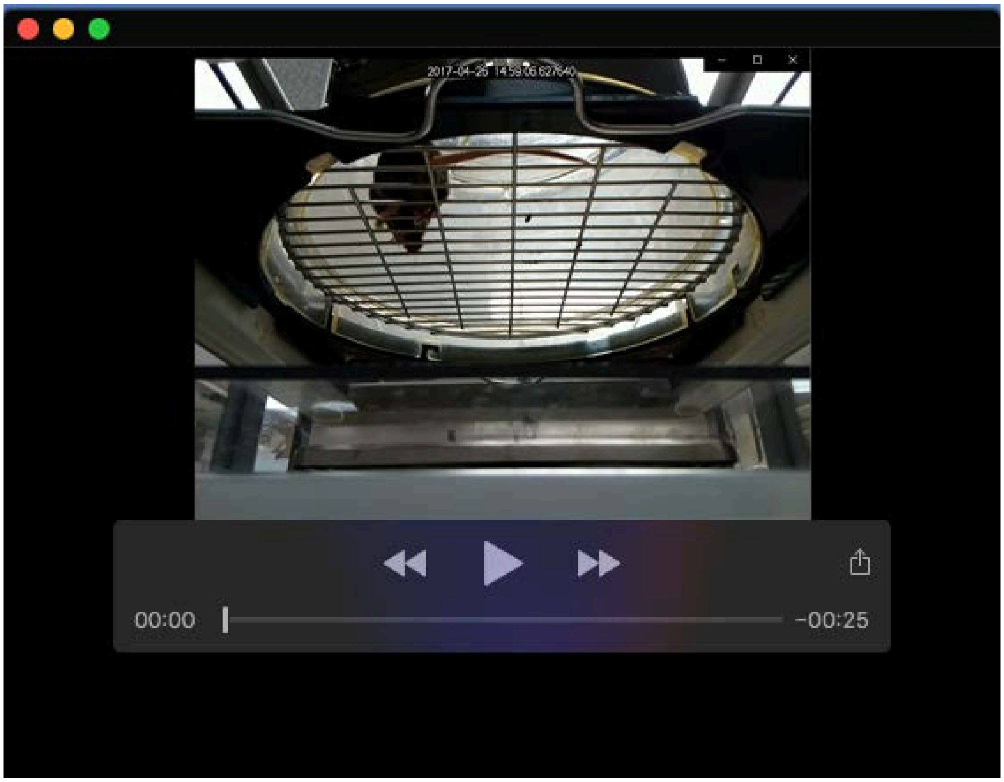

Movie 2. Voiding pattern of KO mouse. This video illustrates the voiding pattern of male mice lacking constitutive expression of *MIR-29a* and *MIR-29b1* that was characterized by expulsion of intermittent droplets of urine.
